# Bandgap Engineering on UiO–66 Metal‐Organic Framework Derivatives for Solar‐Driven Seawater Desalination

**DOI:** 10.1002/advs.202502989

**Published:** 2025-04-11

**Authors:** Qisheng Shao, Yutong Ding, Wenxian Liu, Jia Guan, Ge Meng, Tairong Kuang, Dingsheng Wang

**Affiliations:** ^1^ Functional Polymers & Advanced Materials (FPAM) Lab State Key Laboratory of Advanced Separation Membrane Materials College of Materials Science and Engineering Zhejiang University of Technology Hangzhou 310014 P. R. China; ^2^ Key Laboratory of Carbon Materials of Zhejiang Province College of Chemistry and Materials Engineering Wenzhou University Wenzhou 325035 P. R. China; ^3^ Wuhu Innovation New Materials Co., Ltd Wuhu 241080 P. R. China; ^4^ Department of Chemistry Tsinghua University Beijing 100084 P. R. China

**Keywords:** bandgap engineering, metal‐organic frameworks, photothermal conversion, solar water evaporation, linker

## Abstract

The growing scarcity of freshwater, driven by climate change and pollution, necessitates the development of efficient and sustainable desalination technologies. Solar‐powered interfacial water evaporation has emerged as a promising solution; however, its practical implementation is hindered by the limited availability of efficient and stable photothermal materials. Herein, a bandgap engineering strategy via linker modification to enhance the photothermal conversion capability of metal‐organic frameworks (MOFs) is reported toward efficient solar‐driven desalination. By systematically introducing functional groups with varying electron‐donating and electron‐withdrawing abilities, the energy bandgap of UiO–66–X (X = ─F, ─H, ─OH, ─NH_2_, ─(NH_2_)_2_) is finely tuned. Density functional theory (DFT) calculations and femtosecond transient absorption (fs–TA) spectroscopy reveal that stronger electron‐donating functional groups narrow the bandgap of the MOFs, thereby improving their photothermal conversion efficiency. The optimized UiO–66–(NH_2_)_2_ material reaches a peak surface temperature of 58.7 °C when exposed to simulated sunlight at ≈1 kW·m^−2^ with a photothermal conversion efficiency of 86.50% and an evaporation rate of 2.34 kg·m^−2^·h^−1^ with an evaporation efficiency of 97.40%. This study presents a novel approach for fine‐tuning the bandgap in photothermal materials, offering a pathway toward advanced solar desalination technologies to address the global water scarcity crisis.

## Graphical abstract

This work presents a bandgap engineering strategy via linker modification to enhance the photothermal conversion efficiency of UiO–66 metal‐organic framework (MOF) derivatives, achieving a remarkable evaporation efficiency of 97.40% and an evaporation rate of 2.34 kg·m^−2^·h^−1^ for seawater desalination, thereby contributing to the advancement of sustainable solar‐driven desalination technologies.

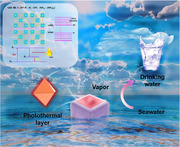

## 1 Introduction

Diminishing freshwater reserves, exacerbated by climate change and pollution, severely impede current sustainable development.^[^ ^1^ ^]^ Although seawater covers more than 96% of the Earth's surface, its direct utilization for daily life is constrained by the lack of efficient desalination technologies. As global demand for freshwater continues to rise, innovative desalination strategies that harness clean energy are drawing increasing attention.^[^ ^2^, ^3^, ^4^, ^5^, ^6^ ^]^ Traditional methods such as reverse osmosis and electrodialysis are widely used but suffer from high energy consumption and environmental concerns, prompting the search for more sustainable alternatives. Among them, solar‐driven desalination has emerged as a promising and sustainable approach that employs photothermal conversion materials (PTMs) to purify seawater by converting solar energy into heat for interfacial evaporation.^[^ ^7^, ^8^, ^9^, ^10^, ^11^, ^12^ ^]^ As the key component of solar desalination systems, advanced PTMs with high stability and efficient photothermal conversion capabilities are of growing interests for their potential to significantly improve desalination performance.

The photothermal conversion efficiency of a material is intrinsically linked to its energy bandgap. A narrower bandgap facilitates the absorption of lower‐energy photons, thereby broadening the spectrum of light that can be harnessed.^[^ ^13^, ^14^, ^15^, ^16^, ^17^, ^18^ ^]^ For example, Tang et al. demonstrated that doping transition metals into graphene narrows its bandgap and enhances photothermal conversion efficiency.^[^ ^19^ ^]^ Bolotin et al. adjusted the band structure of monolayer and bilayer molybdenum disulfide (MoS_2_) through uniaxial tensile strain.^[^ ^20^ ^]^ Similarly, Ogawa et al. substituted Bi^3+^ for Y^3+^ in Bi_2_YO_4_X to narrow the bandgap, offering a potential pathway for photovoltaic applications of transition metal compounds.^[^ ^21^ ^]^ Despite the progress made through these engineering approaches to modulate the bandgap, achieving precise and tunable bandgap control remains a challenge. Therefore, the development of efficient chemical strategies for fine‐tuning the bandgap is urgently needed to optimize photothermal conversion.

Metal‐organic frameworks (MOFs) represent a promising class of materials for photothermal applications owing to their high surface area, structural tunability, and diverse functionalities.^[^ ^22^, ^23^, ^24^, ^25^, ^26^, ^27^ ^]^ Their unique properties, particularly in water transport and evaporation, make them ideal candidates for desalination systems. For instance, Wang et al. developed a flexible Fe–MOF–74‐based composite that achieved a seawater evaporation rate of 1.35 kg·m^−2^·h^−1^ and a solar energy conversion efficiency of 81.5% in seawater desalination.^[^ ^28^ ^]^ Ahmed et al. reported MIL–125 nanomaterials enhanced with graphene, reaching a seawater evaporation rate of 1.26 kg·m^−2^·h^−1^ and a 90% conversion efficiency.^[^ ^29^ ^]^ Selvam et al. coated nickel–doped HKUST–1 (Cu–MOF) onto Janus membranes via high‐temperature calcination, achieving a seawater evaporation rate of 1.30 kg·m^−2^·h^−1^ and a solar energy conversion efficiency of 95.7%.^[^ ^30^ ^]^ Although MOF‐based evaporators show promising performance in desalination, their limited chemical stability hinders long‐term application in marine environments. To overcome this limitation, UiO–66 and its derivatives are often incorporated into multicomponent photothermal materials due to their structural tunability. For example, Xie et al. utilized the photothermal properties of UiO–66@PAN for cancer treatment.^[^ ^31^ ^]^ Almazán et al. converted carbon dioxide (CO_2_) to methane (CH_4_) using the photothermal effect of UiO–66 MOF‐derived Ru@ZrO_2_ catalysts.^[^ ^32^ ^]^ However, pure UiO–66 and its derivatives are rarely used as photothermal materials because their wide intrinsic bandgap limits visible light absorption and facilitates electron‐hole recombination, thereby reducing the utilization of photogenerated carriers.^[^ ^33^ ^]^ This inherent limitation significantly hinders their photothermal conversion efficiency. Therefore, to enable the direct application of UiO–66 in seawater desalination, it is crucial to develop simple and effective strategies that address current limitations in light absorption and photothermal efficiency, which hinder solar energy conversion and evaporation performance.

**Figure 1:**
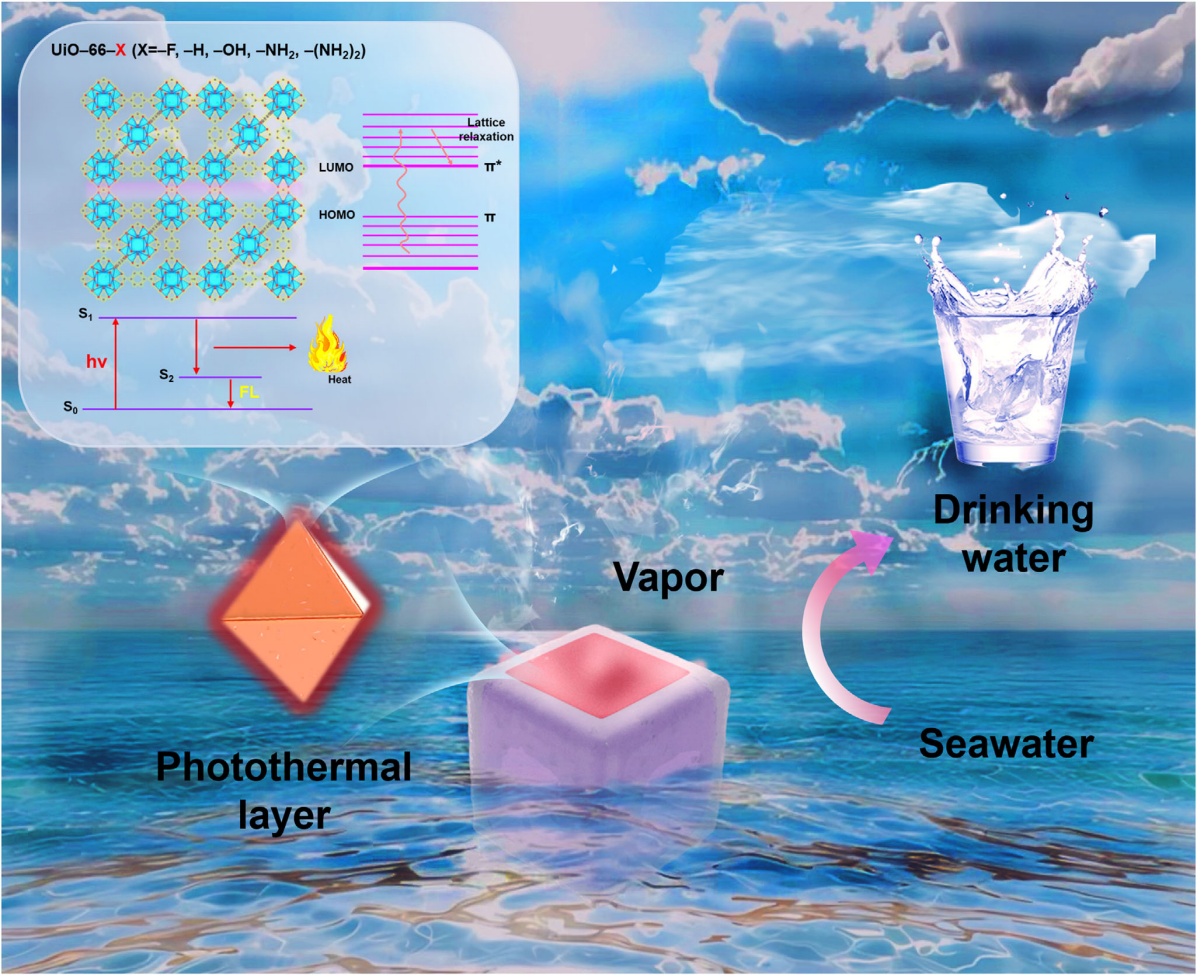
Schematic illustration of the photothermal desalination process using a UiO–66–(NH_2_)_2_‐based‐paper evaporator.

Herein, we propose a bandgap engineering strategy that employs linker modification to fine‐tune the photothermal conversion efficiency of UiO–66 materials. The introduction of functional groups narrows the bandgap, significantly enhancing their photothermal performance and enabling the direct application of pure UiO–66 in efficient solar‐driven seawater desalination (**Figure** **1**). Specifically, by systematically introducing functional groups with varying electron‐donating and electron‐withdrawing properties, the bandgap of UiO–66–X (X = ─F, ─H, ─OH, ─NH₂, ─(NH_2_)_2_) was precisely tuned, resulting in the optimization of the photothermal conversion efficiency from 28.00% to 86.50%. Combining density–functional theory (DFT) calculations with femtosecond transient absorption (fs–TA) spectroscopy, the intrinsic relationship between bandgap modulation, charge carrier dynamics, and photothermal conversion efficiency is disclosed. The optimized UiO–66–(NH_2_)_2_ material, exhibited a remarkable seawater evaporation rate of 2.34 kg·m^−2^·h^−1^ when utilized as a photothermal layer in a water purification device, achieving an evaporation conversion efficiency of 97.40% under solar light exposure, outperforming most reported carbon and MOF‐based systems. This work presents an effective and scalable strategy for bandgap engineering in MOFs, unlocking their potential for high‐performance solar‐driven desalination and broader sustainable energy applications.

## 2 Results and Discussion

**Figure 2:**
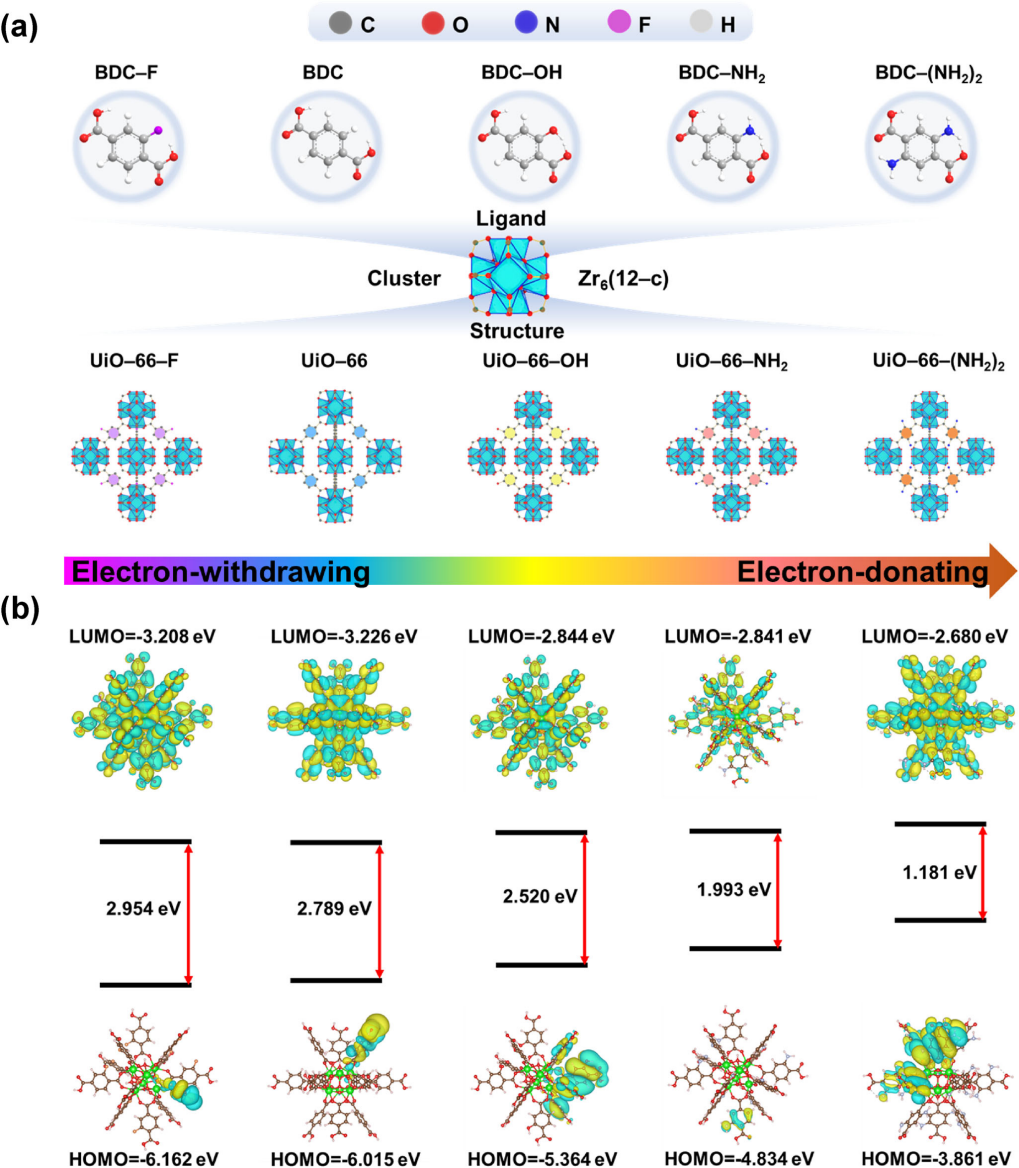
Bandgap modulation of UiO–66–X via linker modification. (a) Schematic representation of the synthesis of UiO–66–X, where X denotes various functional groups (─F, ─H, ─OH, ─NH_2_, ─(NH_2_)_2_). (b) Density–functional theory (DFT) calculations of the HOMO–LUMO energy levels and orbital distributions of UiO–66–X.

Density–functional theory (DFT) calculations were conducted to explore the connection between functional groups (BDC–F, BDC, BDC–OH, BDC–NH_2_, and BDC–(NH_2_)_2_) on the linker (**Figure** **2**a) and energy band structures in UiO–66. As shown in Figure 2b, the highest occupied molecular orbital (HOMO) energy levels of UiO–66–X varied systematically with the electron‐donating ability of the functional groups. For UiO–66–X containing strong electron‐withdrawing groups, such as ─F, the HOMO energy level was significantly lower compared to other functionalized ligands. Conversely, MOFs with strong electron‐donating groups, such as OH, ─NH_2_, and ─(NH_2_)_2_, exhibited a gradual increase in the HOMO energy levels. This trend indicates the influence of electron‐donating groups on the redistribution of electron density within the UiO–66–X framework.^[^ ^34^, ^35^ ^]^ On the other hand, the lowest unoccupied molecular orbital (LUMO) energy levels of UiO–66–X were found to change with the electron‐donating or electron‐withdrawing properties of the functional groups. For UiO–66–F, the LUMO energy level remained nearly constant relative to the unfunctionalized UiO–66. However, in MOFs with strong electron‐donating groups (─OH, ─NH_2_, and ─(NH_2_)_2_), the LUMO energy levels increased progressively, narrowing the energy bandgap. Taken together, these results confirm that the introduction of electron‐withdrawing groups widens UiO–66–X's bandgap, whereas electron‐donating groups lead to bandgap narrowing. Notably, the stronger the capacity of the functional group to give electrons, the narrower the resulting energy bandgap.^[^ ^36^ ^]^

The width of the energy bandgap directly impacts the material's ability to convert light energy into heat. When the photon energy exceeds or matches the energy bandgap, the UiO–66–X nanoparticles undergo photoexcitation, generating photogenerated electrons and holes. During the charge recombination process, these excited electrons cannot effectively radiate the absorbed photon energy, which results in localized lattice heating.^[^ ^37^ ^]^ Additionally, the π–orbital electrons in UiO–66–X can be readily excited from π–orbitals to π^*^–orbitals after absorbing the energy of light. It then undergoes a process of vibration‐electron coupling relaxation, in which the release of excess energy further enhances photothermal conversion, thus returning the excited electrons to the ground state.^[^ ^38^ ^]^ These results indicate that a narrower energy bandgap enhances photothermal conversion efficiency, as smaller bandgaps allow for efficient absorption of incident photons and rapid heat generation.

**Figure 3:**
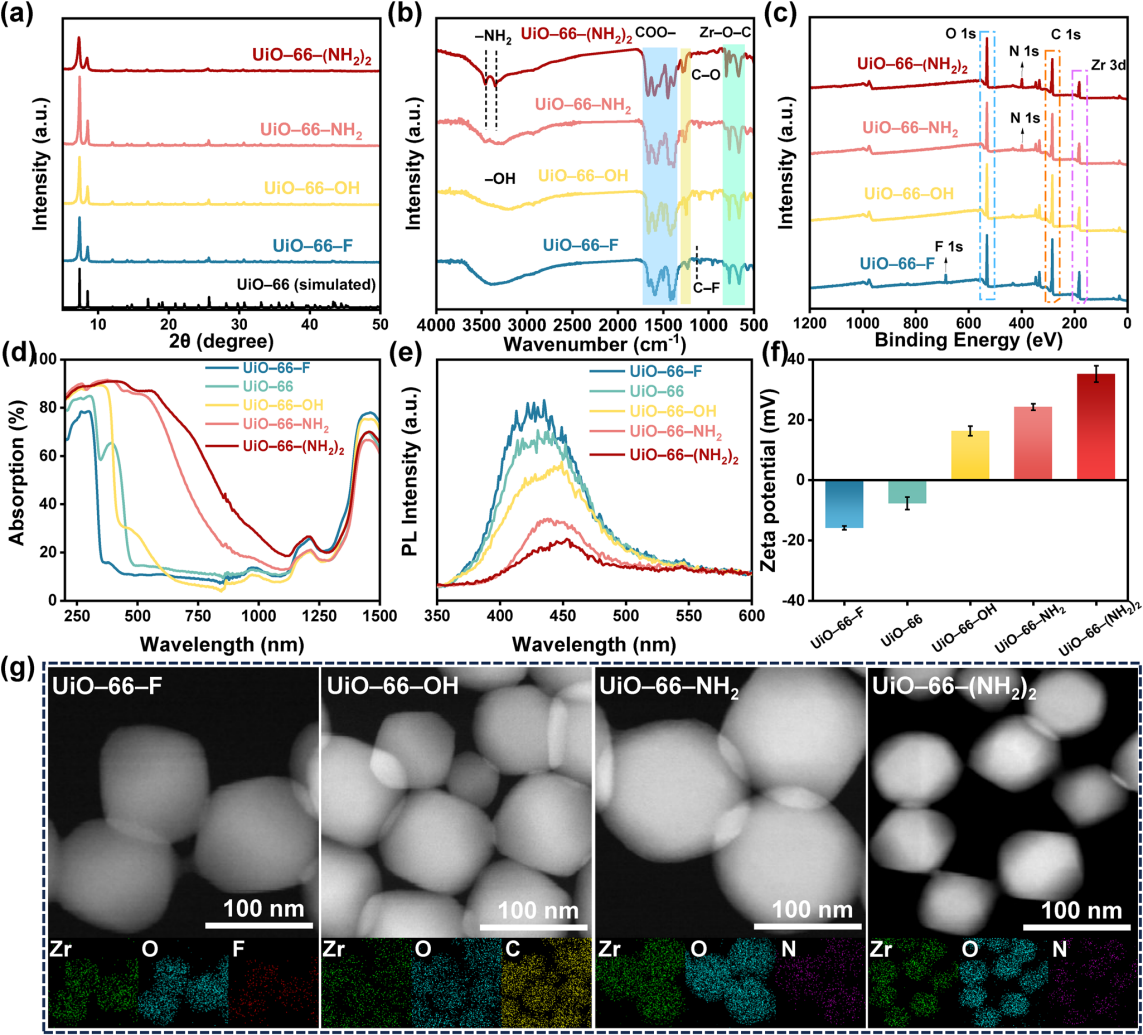
Characterization of UiO–66–X (X = ─F, ─H, ─OH, ─NH_2_, ─(NH_2_)_2_). a) XRD pattern. b) FT–IR spectra. c) XPS spectrogram. d) UV–vis spectrogram (220–1500 nm). e) Fluorescence emission spectrogram. f) Zeta potential analysis. g) TEM images and elemental mappings.

Inspired by the above results, we used BDC–F, BDC, BDC–OH, BDC–NH_2_, and BDC–(NH_2_)_2_ as ligands and coordinated them with Zr^4+^ under solvothermal conditions to synthesize UiO–66–X materials with different electron‐withdrawing and electron‐donating groups, which were named UiO–66–F, UiO–66, UiO–66–OH, UiO–66–NH_2_, and UiO–66–(NH_2_)_2_ (detailed synthesis method provided in the experimental section of Supporting Information). As shown in **Figure** **3**a and Figure S1 (Supporting Information), powder X‐ray diffraction (PXRD) analysis shows that UiO–66 has obvious diffraction peaks at 2θ values of 7.3°, 8.5°, and 25.8°, aligned with (111), (002) and (224) crystal planes, respectively.^[^ ^39^ ^]^ The diffraction pattern of samples carrying different functional groups is consistent with UiO–66,^[^ ^40^, ^41^, ^42^, ^43^ ^]^ indicating that the crystal structure of the framework is not affected by the introduction of functional groups. The presence of functional groups in UiO–66–X was verified through FT–IR spectroscopy (Figure 3b; Figure S2, Supporting Information). All five samples showed distinct Zr–O–C peaks, confirming the successful coordination of Zr^4+^ with the linker. For UiO–66–NH_2_ and UiO–66–(NH_2_)_2_, prominent –NH_2_ stretching vibration peaks were observed near 3500 cm^−1^.^[^ ^44^ ^]^ UiO–66–OH displayed a significantly broader peak near 3500 cm^−1^, attributed to ─OH groups forming hydrogen bonds. This broadening and redshift of the O─H stretching vibration toward lower wavenumbers indicated an elevated ─OH content compared to other samples. Additionally, UiO–66–F exhibited a distinct C─F peak near 1210 cm^−1^.^[^ ^42^ ^]^ These results confirm the successful functionalization of UiO–66 with various ligands.

X‐ray photoelectron spectroscopy (XPS) further validated the incorporation of functional groups (Figure 3c; Figure S6, Supporting Information). The N 1s peak of UiO–66–(NH_2_)_2_ exhibited greater intensity than that of UiO–66–NH_2_, reflecting its higher –NH_2_ content. Similarly, high‐resolution C1s spectra of UiO–66 with different linkers (─F, ─OH, ─NH_2_, and ─(NH_2_)_2_) show peaks corresponding to the C─F, C─O, and C─N bonds near 286.8, 285.9, and 285.2 eV, respectively (Figures S3–S11, Supporting Information).^[^ ^45^, ^46^, ^47^ ^]^ The XPS results of UiO–66–F showed a 0.1 eV reduction in the Zr 3d_3/2_ binding energy relative to UiO–66, which demonstrates the electron absorption effect of the functional group. Due to the electron‐donating properties of ─OH, ─NH_2_, ─(NH_2_)_2_, the binding energies of Zr 3d_3/2_ increased by 0.1, 0.2, and 0.3 eV, respectively. It can be seen that the binding energies of Zr 3d_5/2_ and Zr 3d_3/2_ increase gradually with the increasing electron‐donating ability of the substituent group on the benzene ring.

The light absorption properties of UiO–66–X were analyzed using UV–vis spectroscopy (Figure 3d). The absorbance of UiO–66 in the visible range increased with the electron‐donating ability of the functional groups, as corroborated by calculated light reflectance and transmittance values (Figures S12 and S13, Supporting Information). Fluorescence emission spectra (Figure 3e) revealed that the emission intensity decreased as the bandgap of the MOFs narrowed. This trend indicates that MOFs with smaller bandgaps allocate less energy to fluorescence emission, instead converting more energy into heat.^[^ ^48^ ^]^

Zeta potential measurements provided further insights into the electronic properties of UiO–66–X (Figure 3f). UiO–66–F exhibited a pronounced negative zeta potential, reflecting its strong electron‐withdrawing nature and a tendency to attract electrons, leading to a negatively charged outer surface. In contrast, UiO–66–(NH_2_)_2_ displayed the most positive zeta potential, indicating its strong electron‐donating tendency.^[^ ^49^ ^]^ The morphology of UiO–66–X nanoparticles was analyzed using transmission electron microscopy (TEM) and scanning electron microscopy (SEM) (Figure 3g; Figures S14 and S15, Supporting Information). The particle sizes of UiO–66–F, UiO–66, UiO–66–OH, UiO–66–NH_2_, and UiO–66–(NH_2_)_2_ were ≈200, 100, 180, 250, and 100 nm, respectively. These variations suggest that the morphology and particle size of UiO–66–X are influenced by the functional groups on the linker. The elemental composition of the samples and the homogeneous distribution of the functional groups can be confirmed by energy‐dispersive X‐ray spectroscopy (EDS) mappings.

**Figure 4:**
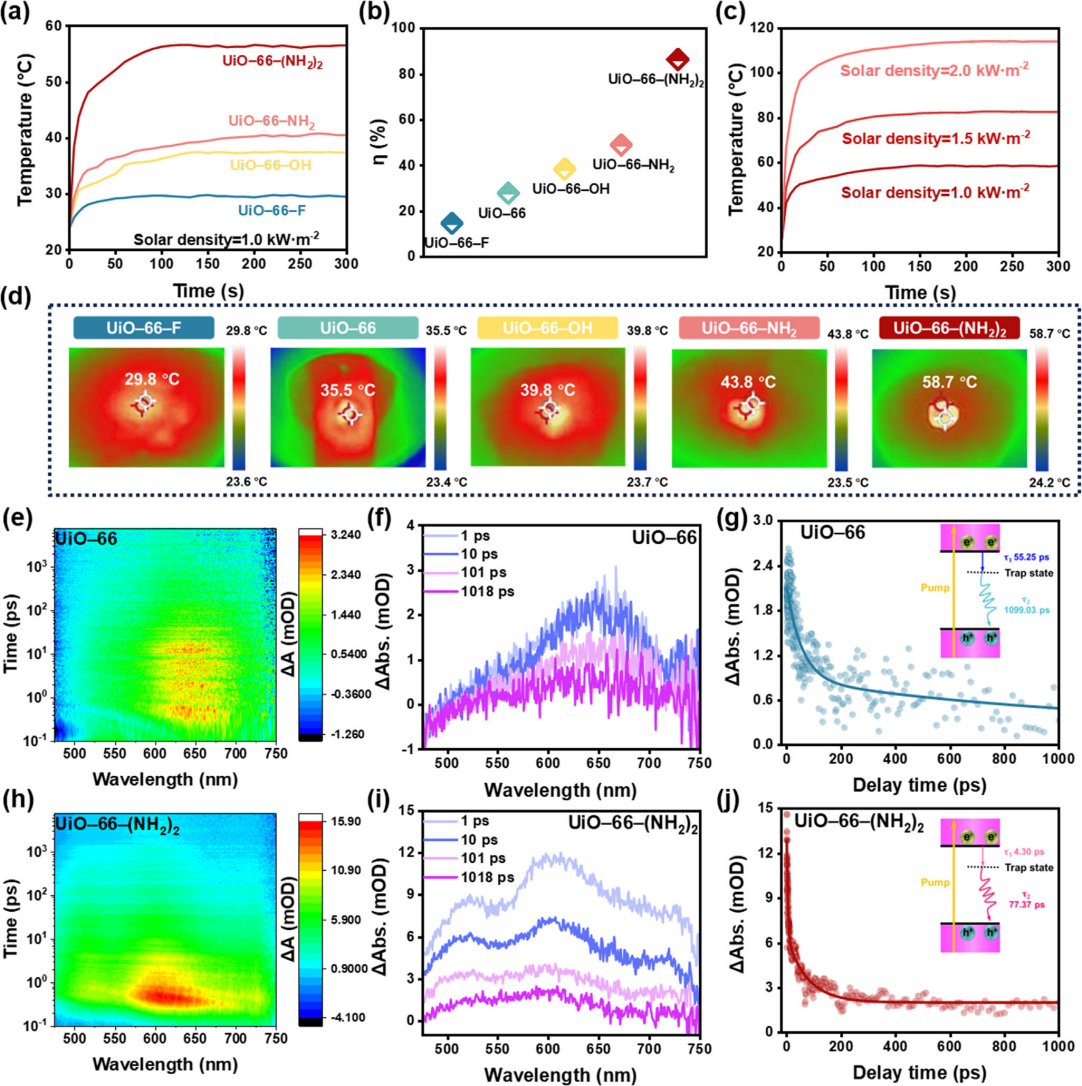
Photothermal performance of UiO–66–X and the impact of bandgap modulation on photothermal conversion capacity (X = ─F, ─H, ─OH, ─NH₂, ─(NH_2_)_2_). a) Surface temperature variations of UiO–66–X over time under one solar intensity (1.0 kW·m^−2^). b) The photothermal conversion efficiency of UiO–66–X at one solar intensity (1.0 kW·m^−2^). c) Surface temperature variation of UiO–66–(NH_2_)_2_ over time under different solar intensities. d) Infrared thermograms of UiO–66–X under one solar intensity. e,f) Transient absorption (TA) spectra and pseudo–color plots for UiO–66 measured at 350 nm excitation with specific delay times. h,i) Transient absorption spectra and pseudo‐color plots for UiO–66–(NH_2_)_2_. g,j) Kinetic decay curves of photogenerated carriers for UiO–66 and UiO–66–(NH_2_)_2_ at 620 nm within 1000 ps.

We used infrared thermography to capture their temperature changes under simulated sunlight (1 kW·m^−2^, 420–2500 nm) irradiation to evaluate the photothermal conversion ability of unsynthesized nanoparticles carrying different functional groups. As illustrated in **Figure** **4**a, the surface temperature of UiO–66–F increased by 5.9 °C, reaching a maximum temperature of 29.8 °C within 300 s, while UiO–66 exhibited a maximum temperature of 35.5 °C (Figure S16, Supporting Information). UiO–66–OH exhibited a temperature rise of 16.0 °C within the same period. The photothermal conversion ability of UiO–66–X modified by –NH_2_ was significantly enhanced by increasing the surface temperature of UiO–66–NH_2_ from 24.2 to 43.8 °C, and that of UiO–66–(NH_2_)_2_ from 24.1 to 58.7 °C. The photothermal conversion efficiency of UiO–66–X at one solar intensity gradually increases as the energy bandgap decreases (detailed calculations provided in Note S1, Supporting Information). Compared with the 28.00% photothermal conversion efficiency of UiO–66, the photothermal conversion efficiency of UiO–66–F with the introduction of an electron‐absorbing functional group decreased by 13.25%, while the energy bandgap of UiO–66–(NH_2_)_2_ with the introduction of a strong electron‐donating functional group was significantly reduced, which led to a substantial increase in the photothermal conversion efficiency to 86.50% (Figure 4b). It is noteworthy that UiO–66–(NH_2_)_2_ still exhibits excellent photothermal conversion ability under different solar intensities, achieving a maximum temperature of ≈58 °C under one solar intensity, which further increased to 82.8 °C at 1.5 kW·m^−2^ and 114.0 °C at 2.0 kW·m^−2^ within 6 min (Figure 4c; Figures S17–S19, Supporting Information).

To assess photothermal stability, cyclic temperature response experiments were conducted on UiO–66–(NH_2_)_2_ under one solar intensity (Figure S20, Supporting Information). The surface temperature stabilized at ≈58 °C for 300 s during each light cycle and quickly returned to room temperature after removal of the light. This cyclic behavior was reproducible over five cycles, demonstrating the excellent photothermal stability and reversibility of UiO–66–(NH_2_)_2_. An analysis of the relationship between the peak surface temperature of UiO–66–X and their bandgap width reveals that a narrower bandgap correlates with the ability to reach a higher surface temperature when exposed to identical sunlight intensity (Figure 4d). To further understand how bandgap variations influence photothermal conversion, the dynamic behavior of ultrafast carriers in UiO–66 and its derivative UiO–66–(NH_2_)_2_ has been meticulously analyzed with the help of femtosecond transient absorption (fs–TA) spectroscopy in this study (Figure 4e,h). The fs–TA contour maps for both samples revealed prominent ground state bleaching (GSB) signals, primarily indicating nonradiative decay pathways for heat generation. For UiO–66, the absorption wavelengths ranged from 600 to 700 nm with a delay time exceeding 10 ps. In contrast, UiO–66–(NH_2_)_2_ exhibited a broader absorption range from 500 to 750 nm and shorter delay times, indicating a rapid and complete vibrational relaxation process enabled by its narrower bandgap. This demonstrates that UiO–66–(NH_2_)_2_ more efficiently converts absorbed light energy into thermal energy. Comparative TA spectra at different delay times are shown in Figure 4f,i. UiO–66 displayed broader positive and negative absorption bands in the 1–1018 ps range (Figure 4f), whereas UiO–66–(NH_2_)_2_ exhibited predominantly positive signals (Figure 4i). The positive signals correspond to excited state absorption (ESA), originating from photogenerated electron‐hole pairs. The narrower range of negative signals in UiO–66–(NH_2_)_2_ indicates a slower carrier recombination rate, allowing more carriers to participate in photothermal conversion.^[^ ^50^ ^]^

The kinetic decay curves of photogenerated carriers at 620 nm provide further insight into the charge carrier dynamics (Figure 4g,j). For UiO–66, the relaxation process was characterized by a fast decay component (τ_1_ = 55.25 ps) and a slower decay component (τ_2_ = 1099.03 ps). The fast decay corresponds to the shallow trap (ST) state, while the slow decay represents long‐lived charge‐separated states involving free electrons and photogenerated holes (Figure 4g). In contrast, UiO–66–(NH_2_)_2_ exhibited significantly reduced time constants (τ_1_ = 4.30 ps, τ_2_ = 77.37 ps), indicating faster charge carrier dynamics and enhanced photothermal conversion efficiency (Figure 4j). These results demonstrate that UiO–66–(NH_2_)_2_, with its narrower bandgap, requires less energy to reach excited states and achieves more efficient light‐to‐heat conversion compared to UiO–66.^[^ ^51^ ^]^ The narrowing of the bandgap facilitates faster carrier dynamics and reduces energy losses through radiative pathways, making UiO–66–(NH_2_)_2_ an excellent candidate for photothermal applications.

**Figure 5:**
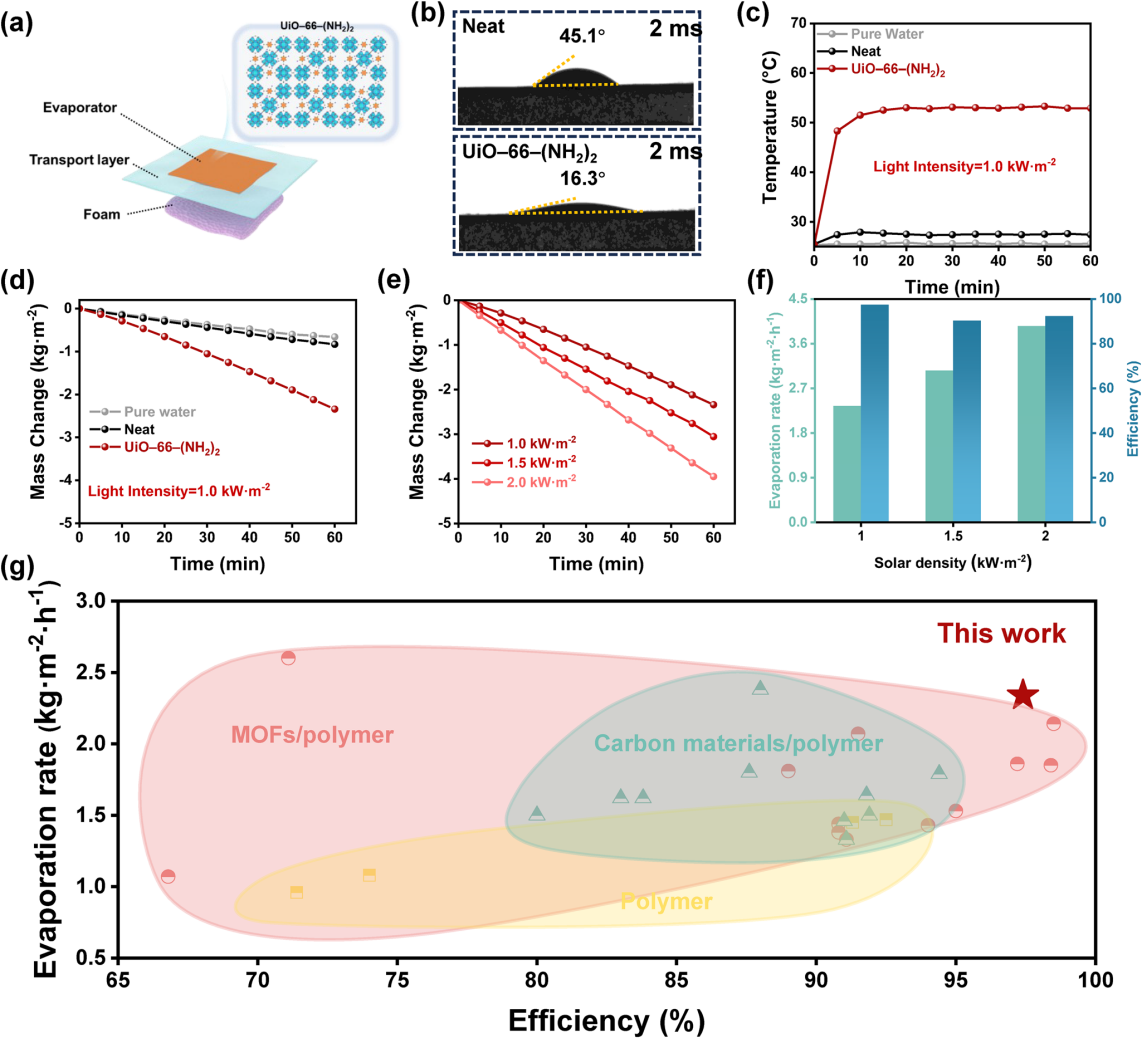
Desalination performance of the UiO–66–(NH_2_)_2_–paper evaporator. a) Schematic illustration of the UiO–66–(NH_2_)_2_–paper evaporator unit. b) Optical photographs of water contact angles for untreated paper and UiO–66–(NH_2_)_2_–paper at 2 ms. c) Surface temperature profiles of water, Paper evaporator, and UiO–66–(NH_2_)_2_–paper evaporator under one solar intensity over time. d) Mass change as a function of time at 1.0 kW·m^−2^ solar intensity. e) Mass change curves over time under varying solar intensities (1.0, 1.5, and 2.0 kW·m^−2^) for the UiO–66–(NH_2_)_2_–paper evaporator. f) Evaporation rate and vapor conversion efficiency of the UiO–66–(NH_2_)_2_–paper evaporator at different solar intensities. g) Comparison of evaporation rates and vapor conversion efficiencies of the UiO–66–(NH_2_)_2_–paper evaporator with previously reported polymer‐based, carbon material/polymer‐based, and MOF/polymer‐based systems.

Solar‐powered desalination is a crucial water recycling technology that relies on high‐performance solar‐harvesting materials to convert solar energy into thermal energy for diverse applications, including seawater desalination and water purification.^[^ ^52^, ^53^, ^54^, ^55^, ^56^, ^57^ ^]^ In this study, the UiO–66–(NH_2_)_2_ material demonstrated exceptional application advantages. By spraying through the spray gun, UiO–66–(NH_2_)_2_ can form a dense and homogeneous photothermal layer on various substrates, imparting superior photothermal properties. Using this material, we constructed a novel desalination device by spraying UiO–66–(NH_2_)_2_ nanoparticles onto cellulose blotting paper. The resulting photothermal‐coated paper was integrated with expanded polystyrene foam to create a lightweight, floating evaporator (**Figure** **5**a). The polystyrene foam gives the entire installation the ability to float on water, while the cellulose paper acts as an efficient water transport layer, mimicking the moisture transport channels found in desert organisms.^[^ ^58^, ^59^, ^60^, ^61^ ^]^ This system ensured continuous water delivery to the photothermal interface for effective evaporation. The UiO–66–(NH_2_)_2_ material, characterized by its alternating hydrophilic and hydrophobic microchannels, played a pivotal role in water transport. The hydrophilic amino groups strongly interacted with water molecules, enhancing water uptake, while the hydrophobic benzene rings reduced resistance during transport, thereby accelerating water flow.^[^ ^62^ ^]^ Contact angle measurements revealed that the UiO–66–(NH_2_)_2_–coated paper exhibited a significantly lower contact angle (16.3°) compared to uncoated paper (45.1°) (Figure 5b), confirming its superior hydrophilicity and efficient water transport capability. To evaluate the photothermal performance of the device, a xenon lamp, calibrated to simulate sunlight, was employed as the light source (Figure 5c). Under 1.0 kW·m^−2^ solar intensity, the UiO–66–(NH_2_)_2_–paper evaporator's surface temperature rapidly increased, reaching a maximum equilibrium temperature of≈53 °C within 20 min (Figure S21, Supporting Information). In contrast, the uncoated paper evaporator achieved a maximum temperature of only≈27.8 °C. This significant temperature difference underscores the superior photothermal conversion efficiency of UiO–66–(NH_2_)_2_, which is critical for accelerating water evaporation under simulated sunlight. Real‐time monitoring of the device's mass change during evaporation confirmed the enhanced performance of the UiO–66–(NH_2_)_2_‐based evaporator (Figure 5d–f). Evaporation rates of 2.34, 3.06, and 3.95 kg·m^−2^·h^−1^ were achieved at solar radiation intensities of 1, 1.5, and 2 kW·m^−2^. After subtracting the dark–field evapotranspiration, the evaporative conversion efficiencies at these intensities were 97.40%, 90.29%, and 92.29%, respectively (detailed calculations provided in Notes S2–S4, Supporting Information).^[^ ^62^, ^63^ ^]^ Moreover, the UiO–66–(NH_2_)_2_ exhibits excellent long‐term stability. Comprehensive photostability and seawater evaporation stability tests (Figures S22–S26, Supporting Information) confirmed that on UiO–66–(NH_2_)_2_ maintained its original morphology and structural integrity after 168 h of continuous light exposure. Its evaporation performance remained stable, with the UiO–66–(NH_2_)_2_–paper evaporator consistently achieving a rate of ≈2.30 kg·m^−2^·h^−1^ without significant degradation. Long‐term immersions in simulated seawater further confirmed its sustained and efficient photothermal performance. These results highlight the outstanding photothermal efficiency of UiO–66–(NH_2_)_2_ in solar‐driven desalination.

The results of the comparative study further validate that UiO–66–(NH_2_)_2_‐based materials exhibit outstanding performance compared to conventional polymer materials, carbon/polymer composites and MOF/polymer systems (Figure 5g; Tables S1–S3, Supporting Information). When exposed to a solar intensity of 1.0 kW·m^−2^, the evaporator constructed from UiO–66–(NH_2_)_2_‐based evaporator demonstrated an evaporation rate of 2.34 kg·m^−2^·h^−1^, along with an evaporation conversion efficiency of 97.40%, outperforming most previously reported materials. These results confirm that UiO–66–(NH_2_)_2_ not only enhances water transport and photothermal conversion but also sets a new benchmark for high–performance, solar‐driven desalination systems.

To comprehensively evaluate the practical application potential of the UiO–66–(NH_2_)_2_–paper evaporator, a prototype was carefully designed and fabricated. Comparative experiments were conducted using a control evaporator composed of paper without a photothermal layer. The experiments were carried out at the Moganshan Campus of Zhejiang University of Technology in Huzhou, Zhejiang Province, China (30.33 °N, 120.2 °E). The evaporation performance of the device was evaluated in various water environments, including simulated seawater, real seawater, and lake water. Detailed compositions are provided in Table S4 (Supporting Information).

**Figure 6:**
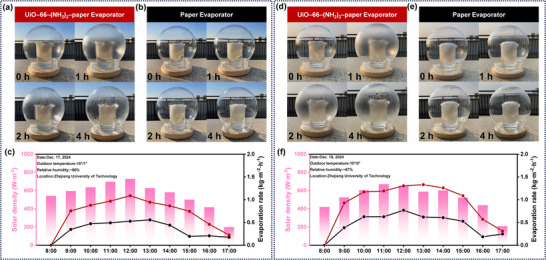
Outdoor evaporation experiments using different water sources. Simulated seawater: a) Optical photographs showing the UiO–66–(NH_2_)_2_–paper evaporator at varying daylight intervals (0, 1 , 2 , and 4 h). b) Optical photographs of the Paper evaporator under identical daylight conditions. c) Variation in solar intensity and evaporation rates of the UiO–66–(NH_2_)_2_–paper evaporator and Paper evaporator during outdoor testing on a sunny day from 8:00 to 17:00. Real seawater: d) Optical photographs of the UiO–66–(NH_2_)_2_–paper evaporator under different durations of sunlight exposure. e) Optical photographs of the Paper evaporator during the same exposure periods. f) Cyclic variation of solar intensity and evaporation rates of the UiO–66–(NH_2_)_2_–paper evaporator and Paper evaporator on a clear day from 8:00 to 17:00.

The desalination performance of the UiO–66–(NH_2_)_2_–paper evaporator was first evaluated using artificial seawater with a salt content of 3.4% (**Figure** **6**a,b; Figure S27, Supporting Information). Under natural sunlight, a uniform layer of water mist rapidly formed on the inner glass dome of the UiO–66–(NH_2_)_2_–paper evaporator within 1 h. In contrast, the control Paper evaporator showed no mist condensation even after an hour of sunlight exposure. By the end of 4 h, the UiO–66–(NH_2_)_2_–paper evaporator exhibited significant water droplet condensation on the glass dome, which could be effectively collected, whereas the control remained wet without sufficient droplet formation. Throughout the experiment, detailed weather parameters, including sunlight intensity and relative humidity, were recorded (Figure 6c). Moisture evaporation from the UiO–66–(NH_2_)_2_–paper evaporator at different times of the day exhibited a strong positive correlation with sunlight intensity, reaching peak performance during midday when solar intensity was at its highest. This confirmed the stable and efficient operation of the evaporator under natural conditions. To validate the robustness of the device, its evaporation performance was further tested in real seawater (Figures 6d,e; Figure S28,Supporting Information). After 2 h of sunlight exposure, the UiO–66–(NH_2_)_2_–paper evaporator facilitated the formation of numerous small water droplets on the glass dome, which were easily collected. In contrast, the Paper evaporator without a photothermal layer exhibited significantly inferior performance. Over the course of a day, evaporation from the UiO–66–(NH_2_)_2_–paper evaporator maintained a positive correlation with the intensity of sunlight (Figure 6f), demonstrating its consistent performance in seawater. The versatility of the UiO–66–(NH_2_)_2_–paper evaporator was further investigated by testing it in river water environments (Figures S29–S32, Supporting Information). The results revealed a similar trend: the evaporation rate increased with solar intensity, confirming the device's applicability across various water sources. The evaporator consistently delivered high evaporation performance, and the pure water generated during the process was efficiently collected (Figure S33, Supporting Information).

The results clearly establish the UiO–66–(NH_2_)_2_–paper evaporator as a versatile and efficient solution for solar‐driven water evaporation in diverse outdoor environments. Its ability to perform consistently in simulated seawater, real seawater, and lake water underscores its adaptability. Furthermore, the efficient condensation and collection of pure water make it highly practical for real‐world desalination and water purification applications.

## 3 Conclusion

In summary, we developed a bandgap engineering strategy to enhance the photothermal conversion efficiency of UiO–66 materials by systematically introducing electron‐donating and electron‐withdrawing functional groups. This approach effectively addresses the intrinsic limitations of pure UiO–66, including its wide bandgap and high electron‐hole recombination rate, thereby enabling its direct application in solar‐driven desalination. Impressively, the optimized UiO–66–(NH_2_)_2_ exhibited a remarkable evaporation rate of 2.34 kg·m^−2^·h^−1^ along with an outstanding evaporation efficiency of 97.40% for seawater desalination, surpassing most existing carbon‐ and MOF‐based systems. The intrinsic mechanism of bandgap modulation was elucidated through the mutual corroboration of DFT calculations and fs–TA spectroscopy, providing important insights into the relationship between electronic structure, charge‐carrier dynamics, and photothermal performance. This work not only offers a new approach to the fine‐tuning of photothermal materials but also contributes to the advancement of sustainable seawater desalination technologies.

## Conflict of Interest

The authors declare no conflict of interest.

## Author Contributions

Q.S., T.K., W.L., G.M., and D.W. performed conceptualization; Q.S., Y.D., and J.G. performed methodology and investigation; T.K. performed supervision; T.K., W.L., G.M., and D.W. wrote the original draft reviewed and edited the final manuscript.

## Supporting information



Supporting Information

## Data Availability

The data that support the findings of this study are available from the corresponding author upon reasonable request.
