# Supplementary material for: Bandgap Engineering on UiO–66 Metal‐Organic Framework Derivatives for Solar‐Driven Seawater Desalination
Source: Adv Sci (Weinh). 2025 Apr 11;12(26):2502989. doi: 10.1002/advs.202502989 (PMC12245057; doi:10.1002/advs.202502989)
Supplement: Supplementary file 1 — Supporting Information [file ADVS-12-2502989-s001.docx]

**Supporting Information**

**Bandgap Engineering on UiO–66 Metal-Organic Framework Derivatives for Solar-Driven Seawater Desalination**

Qisheng Shao, Yutong Ding, Wenxian Liu^*^, Jia Guan, Ge Meng^*^, Tairong Kuang^*^, Dingsheng Wang^4^

Q. Shao, Y. Ding, W. Liu, G. Meng, T. R. Kuang

Functional Polymers & Advanced Materials (FPAM) Lab, Zhejiang Key Laboratory of Plastic Modification and Processing Technology, College of Materials Science and Engineering, Zhejiang University of Technology, Hangzhou 310014, P. R. China.

E-mail: [kuangtr@zjut.edu.cn](mailto:kuangtr@zjut.edu.cn); [liuwx@zjut.edu.cn](mailto:liuwx@zjut.edu.cn).

T. R. Kuang

Wuhu Innovation New Materials Co., Ltd, Wuhu 241080, P. R. China.

J. Guan, G. Meng

^3^ Key Laboratory of Carbon Materials of Zhejiang Province, College of Chemistry and Materials Engineering, Wenzhou University, Wenzhou 325035, P. R. China.

Email: [mengge@wzu.edu.cn](mailto:mengge@wzu.edu.cn).

D. S. Wang

Department of Chemistry, Tsinghua University, Beijing 100084, China.

***Supplementary Experimental Section***

***Materials***

Zirconium chloride (100 g, Shuangmu Chemical, Hangzhou, China), sodium chloride (100 g, Shuangmu Chemical, Hangzhou, China), magnesium chloride (500 g, Shuangmu Chemical, Hangzhou, China) potassium sulphate (500 g, Shuangmu Chemical, Hangzhou, China), and calcium carbonate (100 g, Shuangmu Chemical, Hangzhou, China) were purchased from the brand of Macklin. 2–fluoroterephthalic acid ( Ltd., Shanghai, China), terephthalic acid (25 g, Marel Biochemical Technology Co., Ltd., Shanghai, China), 2–hydroxyterephthalic acid (1 g, Marel Biochemical Technology Co., Ltd., Shanghai, China), 2–amino–terephthalic acid (5 g, Marel Biochemical Technology Co., Ltd., Shanghai, China) and 2,5–diaminoterephthalic acid (5 g, Marel Biochemical Technology Co., Ltd., Shanghai, China) were purchased from MACKLIN brand. acid (5 g, Meryer Biochemical Technology Co., Ltd, Shanghai, China) were purchased from the Meryer brand. Anhydrous ethanol (4 L, Bangyi Chemical, Hangzhou, China), N, N–dimethylformamide (500 mL, Sinopharm Group, Beijing, China) and glacial acetic acid (500 mL, Jigong Bio, Hangzhou, China) were purchased from Aladdin brand. Cellulose absorbent paper (thickness **~**180 μm, Huichuang Instrument and Equipment Co., Ltd, Hangzhou, China) was purchased from Vinda brand. Polystyrene foam blocks (5 cm × 5 cm × 3 cm, 0.57 g) were purchased from Youchuang Packaging Technology Co. The real seawater is taken from Lingshan Bay, Huangdao District, Qingdao City, Shandong Province, and the lake water is taken from the Moganshan Campus of Zhejiang University of Technology, Deqing County, Huzhou City, Zhejiang Province. Deionized water was used for all experiments. All chemicals were obtained from commercial suppliers and used without further purification.

***Synthesis of UiO–66–X***

UiO–66–X was synthesized by modification on the basis of literature reports.^[1]^ 150 mg ZrCl_4_, BDC–X (X= –F, –H, –OH, –NH_2_, –(NH_2_)_2_), 15 mL DMF, 6 mL CH_3_COOH and 2 mL deionized water were added to a 50 mL round-bottomed flask. The amounts of 2–fluoro–terephthalic acid (BDC–F), terephthalic acid (BDC), 2–hydroxy terephthalic acid (BDC–OH), 2–amino–terephthalic acid (BDC–NH_2_) and 2, 5–diamino–terephthalic acid (BDC–(NH_2_)_2_) were 178 mg, 146 mg, 178 mg, 176 mg and 187 mg, respectively. After dissolution by sonication, leave in an oil bath at 150 °C for 24 h. After cooling to room temperature, it was washed three times with DMF and ethanol, and then dried in an oven at 60 °C.

***Preparation of the Light–Absorbing Materials***

200 mg of UiO–66–(NH_2_)_2_ powder was added to 10 mL of anhydrous ethanol and sonicated for 30 minutes to ensure uniform dispersion. The UiO–66–(NH_2_)_2_ suspension (0.02 g/mL) was uniformly sprayed onto square cellulose blotting paper (~180 µm thick) using a spray gun. The coated papers were vacuum-dried at 30 °C for 2 h. The coated paper was wrapped around a 5 cm x 5 cm x 3 cm block of expanded polystyrene (0.97 g).

***Preparation of simulated seawater***

Simulated seawater with a salinity of 3.4 % was prepared by dissolving NaCl (27.23 g), MgCl_2_ (10.78 g), CaCO_3_ (1.00 g), and K_2_SO_4_ (0.89 g) in 1 L of deionized water, followed by stirring for 1 h.

***Solar Water Evaporation***

The effective area of the evaporation layer was 8.4 cm^2^. The evaporator was exposed to varying intensities of simulated sunlight for 60 min, with mass changes monitored in real time using an electronic balance. Surface temperature changes were recorded using an infrared thermometer. All experiments were conducted indoors at 25 °C with a relative humidity of ~60%.

***Characterizations***

The morphology of UiO–66–X was analyzed using field emission scanning electron microscopy (FE–SEM, HITACHI Regulus 8100, HITACHI, Japan) and transmission electron microscopy (TEM, Tecnai G2 F30 Stwin, Philips–FEI, Netherlands). UV–vis–NIR spectra were recorded using a UV–vis–NIR spectrophotometer (Lambda 950, PerkinElmer, USA). X-ray diffraction (XRD) patterns were collected using an X-ray diffractometer (X’Pert Pro, PANalytical, Netherlands) with a scanning range of 5°–50° and a scanning speed of 10°/min. Fourier transform infrared spectroscopy (FTIR) was conducted in reflectance (ATR) mode using an FTIR spectrometer (Nicolet iS50, Thermo Fisher, USA) over the range of 4000–400 cm^-1^. X-ray photoelectron spectroscopy (XPS) was performed using a K-Alpha instrument (Thermo Scientific, USA). Zeta potential measurements were conducted using dynamic light scattering (DLS, Brookhaven Omnis, USA). Contact angles were measured using a dynamic contact angle tester (LAUDA LSA100, Germany). Thermal images were captured using a thermal imaging camera (HIKMICRO HM–TPH11–3AXF). Mass changes during evaporation were monitored using an electronic balance (MS105DU, Mettler Toledo, USA). Light intensity was calibrated using an optical irradiator (TES–132, Taiwan). Simulated sunlight was provided by a solar simulator (CME–SL500, China). Transient species properties under 350 nm laser excitation were analyzed using a femtosecond transient absorption spectrometer (fs–TAS, Legend Elite–1K–HE). Information on the chemical composition of the water used in the outdoor experiments was analyzed using an ion chromatograph (Thermo Scientific ICS5000+, USA).

***Density functional theory (DFT) calculations***

All the calculations are performed in the framework of the density functional theory with the projector augmented plane-wave method, as implemented in the Vienna ab initio simulation package.^[2]^ The generalized gradient approximation proposed by Perdew-Burke-Ernzerhof (PBE) is selected for the exchange-correlation potential.^[3]^ The cut-off energy for the plane wave is set to 480 eV. The energy criterion is set to 10^-4^ eV in the iterative solution of the Kohn–Sham equation. All the structures are relaxed until the residual forces on the atoms have declined to less than 0.05 eV/Å. To avoid interlaminar interactions, a vacuum spacing of 20 Å is applied perpendicular to the slab.

***Supplementary Note***

***Supplementary Note S1: Calculation of photothermal conversion efficiency***

The photothermal conversion efficiency is defined as the ratio of the energy gained by the evaporator ($\text{Q}_{\text{in}}$) to the solar radiant energy ($\text{R}_{\text{s}}$) and is calculated by the formula

$$\begin{aligned} \text{η}_{\text{p}}\text{=}\frac{\text{Q}_{\text{in}}}{\text{R}_{\text{s}}}\#\left( \text{S1} \right) \end{aligned}$$

When sunlight strikes the MOF, the temperature of the MOF surface rises due to the photothermal effect. When the temperature of the sample surface is higher than the surrounding ambient temperature, some of the energy is transferred to the environment. When the temperature is constant, the energy lost ($\text{Q}_{\text{out}}$) is equal to the energy produced by solar radiation. Therefore, it can be concluded that $\text{Q}_{\text{in}}\text{=}\text{Q}_{\text{out}}$.

The lost energy ($\text{Q}_{\text{out}}$) is given by

$$\begin{aligned} \text{Q}_{\text{out}}\text{=S}\text{h}_{\text{c}}\left( \text{T}_{\text{1}}\text{–}\text{T}_{\text{0}} \right)\text{t}\#\left( \text{S2} \right) \end{aligned}$$

where $\text{S}$ is the area of the aperture. $\text{h}_{\text{c}}$ is the integrated heat transfer coefficient (25 W·m^-2^·K^-1^). $\text{T}_{\text{1}}$ is the maximum surface temperature of the MOF after constancy. $\text{T}_{\text{0}}$ is the initial surface temperature of the MOF.

Therefore, the photothermal conversion efficiency ($\text{η}_{\text{p}}$) of MOF is calculated as

$$\begin{aligned} \text{η}_{\text{p}}\text{=}\frac{\text{Q}_{\text{out}}}{\text{S}\text{q}_{\text{i}}\text{t}}\text{=}\frac{\text{h}_{\text{c}}\left( \text{T}_{\text{1}}\text{–}\text{T}_{\text{0}} \right)}{\text{q}_{\text{i}}}\#\left( \text{S3} \right) \end{aligned}$$

where $\text{q}_{\text{i}}$ is the solar intensity (1 kW m^–2^ ).

The parameters and results of the calculations are shown below.

| MOF | $\text{T}_{\text{1}}$(K) | $\text{T}_{\text{0}}$(K) | $\text{h}_{\text{c}}\left( \text{T}_{\text{1}}\text{–}\text{T}_{\text{0}} \right)$(W·m^-2^) | $\text{η}_{\text{p}}$(%) |
| --- | --- | --- | --- | --- |
| UiO–66–F | 302.95 | 297.05 | 147.50 | 14.75 |
| UiO–66 | 308.65 | 297.45 | 280.00 | 28.00 |
| UiO–66–OH | 312.95 | 297.55 | 385.00 | 38.50 |
| UiO–66–NH_2_ | 316.95 | 297.35 | 490.00 | 49.00 |
| UiO–66–(NH_2_)_2_ | 331.85 | 297.25 | 865.00 | 86.50 |

***Supplementary Note S2: Calculation of Enthalpy of Evaporation***

***1. Evaporation in dark environments***

1.1. Estimate the enthalpy of evaporation in a dark environment. Here, we assume that the solar evaporator and pure water receive the same energy from the outside world in a dark environment. The details are as follows. Place the same area of the evaporator and pure water. Place the evaporator and the same area of pure water on an electronic balance in a dark environment and record the mass change after 1 h. The room temperature here is 25 °C and the ambient humidity is about 60%. In this environment, the room temperature is 25 °C and the ambient humidity is about 60 %. The enthalpy of evaporation of an evaporator in a dark environment is calculated as

$$\begin{aligned} \text{m}_{\text{w,25 °C}}\text{h}_{\text{v}\text{,w,25 °C}}\text{=}\text{m}_{\text{e,25 °C}}\text{h}_{\text{v,e,25 °C}}\#\left( \text{S4} \right) \end{aligned}$$

where $\text{m}_{\text{w,25 °C}}$ and $\text{m}_{\text{e,25 °C}}$ are the mass change per unit time in the dark for pure water and evaporator, respectively. $\text{h}_{\text{v}\text{,w,25 °C}}$ and $\text{h}_{\text{v,e,25 °C}}$ are the enthalpies of evaporation at 25 °C for pure water and evaporator, respectively. In this experiment, $\text{m}_{\text{w,25 °C}}$ is 0.2635 kg·m^-2^·h^-1^ and $\text{m}_{\text{e,25 °C}}$ is 0.3724 kg·m^-2^·h^-1^. And $\text{h}_{\text{v}\text{,w,25 °C}}$ is 2453.3 J·g^-1^. Therefore, $\text{h}_{\text{v,e,25 °C}}$ is 1662.24 J·g^-1^.

1.2. estimate the enthalpy of evaporation at different temperatures. The enthalpy of evaporation of pure water at different temperatures is given by

$\begin{aligned} \text{h}_{\text{v,w,}\text{T}_{\text{1}}}\text{=}\text{C}_{\text{l}}\left( \text{373.15–}\text{T}_{\text{1}} \right)\text{+}\text{h}_{\text{v,w,100 °C}}\text{+}\text{C}_{\text{g}}\left( \text{T}_{\text{1}}\text{–373.15} \right)\#\left( \text{S5} \right) \end{aligned}$where $\text{h}_{\text{v,w,}\text{T}_{\text{1}}}$ is the enthalpy of evaporation of pure water at temperature $\text{T}_{\text{1}}$. $\text{T}_{\text{1}}$ is the temperature of the evaporator and the surface of the pure water. $\text{C}_{\text{l}}$ is the specific heat capacity of liquid water (4.2 J·K^-1^·g^-1^). $\text{h}_{\text{v,w,100 °C}}$ is the enthalpy of evaporation of pure water at 100 °C (2257 J·g^-1^). $\text{C}_{\text{g}}$ is the specific heat capacity of gaseous water, and the formula for $\text{C}_{\text{g}}$ is

$$\begin{aligned} \text{C}_{\text{g}}\text{=}\left( \text{3.470+1.45×}\text{10}^{\text{-}\text{3}}\text{×}\text{T}_{\text{1}}\text{+0.121×}\text{10}^{\text{5}}\text{×}\text{T}_{\text{2}} \right)\text{R}\text{M}^{\text{-}\text{1}}\#\left( \text{S6} \right) \end{aligned}$$

where $\text{R}$ is the general gas constant (8.314 J·K^-1^·mol^-1^) and $\text{M}$ is the relative molecular mass of water (18.02 g·mol^-1^). By the deformed substitution of Eqs. S4, S5 and S6, the estimation of the enthalpy of evaporation for the UiO–66–(NH_2_)_2_–paper evaporator at different temperatures can be given as

$$\begin{aligned} \text{h}_{\text{v,e,}\text{T}_{\text{1}}}\text{=}\frac{\text{m}_{\text{w,25 °C}}}{\text{m}_{\text{e,25 °C}}}\text{h}_{\text{v,w,}\text{T}_{\text{1}}}\#\left( \text{S7} \right) \end{aligned}$$

For the UiO–66–(NH_2_)_2_–paper evaporator the parameters were calculated as follows.

| Light intensity (kW·m^-2^) | $\text{T}_{\text{1}}$ (K) | $\text{h}_{\text{v,w,}\text{T}_{\text{1}}}$(J·g^-1^) | $\text{h}_{\text{v,e,}\text{T}_{\text{1}}}$(J·g^-1^) |
| --- | --- | --- | --- |
| 1 | 326.45 | 2365.50 | 1662.24 |
| 1.5 | 339.55 | 2334.77 | 1640.65 |
| 2 | 356.45 | 2295.47 | 1613.03 |

**Supplementary note S3: Calculation of enthalpy change**

For the enthalpy change during water evaporation in the UiO–66–(NH_2_)_2_–paper evaporator, the formula is

$$\begin{aligned} \text{h}_{\text{lv,e,}\text{T}_{\text{1}}}\text{=}\text{C}_{\text{l}}\text{×}\left( \text{T}_{\text{1}}\text{–}\text{T}_{\text{0}} \right)\text{+}\text{h}_{\text{v,e,}\text{T}_{\text{1}}}\#\left( \text{S8} \right) \end{aligned}$$

The parameters and calculations are as follows.

| $\text{T}_{\text{1}}$ (K) | $\text{T}_{\text{0}}$ (K) | $\text{C}_{\text{l}}\text{×}\left( \text{T}_{\text{1}}\text{–}\text{T}_{\text{0}} \right)$ | $\text{h}_{\text{v,e,}\text{T}_{\text{1}}}$(J·g^-1^) | $\text{h}_{\text{v,e,}\text{T}_{\text{1}}}$(J·g^-1^) |
| --- | --- | --- | --- | --- |
| 326.45 | 298.45 | 117.60 | 1662.24 | 1779.84 |
| 339.55 | 298.65 | 171.78 | 1640.65 | 1812.43 |
| 356.45 | 298.55 | 243.18 | 1613.03 | 1856.21 |

**Supplementary note S4: Calculation of solar water–steam conversion efficiency**

The solar water evaporation conversion efficiency ($\text{η}_{\text{e}}$) is calculated by the formula

$$\begin{aligned} \text{η}_{\text{e}}\text{=}\frac{\text{m}\text{h}_{\text{lv,e,}\text{T}_{\text{1}}}}{\text{q}_{\text{i}}}\#\left( \text{S9} \right) \end{aligned}$$

Substituting Equation S5 into Equation S6 yields

$$\begin{aligned} \text{η}_{\text{e}}\text{=}\frac{\text{m}\left[ \text{C}_{\text{l}}\text{×}\left( \text{T}_{\text{1}}\text{–}\text{T}_{\text{0}} \right)\text{+}\text{h}_{\text{v,e,}\text{T}_{\text{1}}} \right]}{\text{q}_{\text{i}}}\#\left( \text{S10} \right) \end{aligned}$$

where $\text{m}$ is the net evaporation rate minus the dark evaporation rate of the evaporator at different light intensities. $\text{C}_{\text{l}}$ is the specific heat capacity of water (4.2 J·g^-1^·K^-1^ ). $\text{h}_{\text{lv,e,}\text{T}_{\text{1}}}$ is the enthalpy of evaporation of water at $\text{T}_{\text{1}}$ temperature. $\text{h}_{\text{v,e,}\text{T}_{\text{1}}}$ is UiO–66–(NH_2_)_2_–paper enthalpy of evaporation of the evaporator at temperature $\text{T}_{\text{1}}$, as detailed in S7. $\text{q}_{\text{i}}$ is the light intensity (1, 1.5, 2 kW·m^-2^ ). $\text{T}_{\text{1}}$ is the surface temperature of the evaporator.

The parameters and calculations at different light intensities are given below.

| $\text{T}_{\text{1}}$ (K) | $\text{m}$ (kg·m^-2^·h^-1^) | $\text{C}_{\text{l}}\text{×}\left( \text{T}_{\text{1}}\text{–}\text{T}_{\text{0}} \right)$ | $\text{h}_{\text{lv,e,}\text{T}_{\text{1}}}$(J·g^-1^) | $\text{η}_{\text{e}}$(%) |
| --- | --- | --- | --- | --- |
| 326.45 | 1.97 | 117.60 | 1779.84 | 97.40 |
| 339.55 | 2.69 | 171.78 | 1812.43 | 90.29 |
| 356.45 | 3.58 | 243.18 | 1856.21 | 92.29 |

***Supplementary Figures***


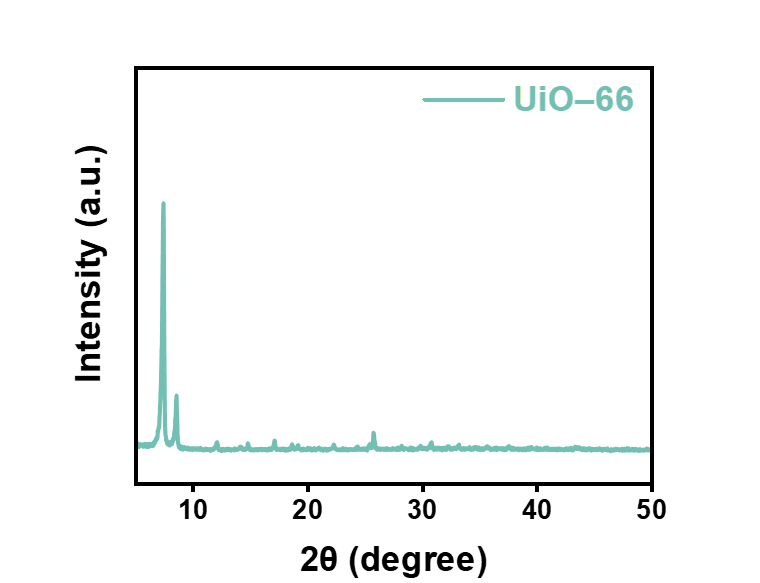


**Supplementary Figure S1.** XRD pattern of UiO–66.


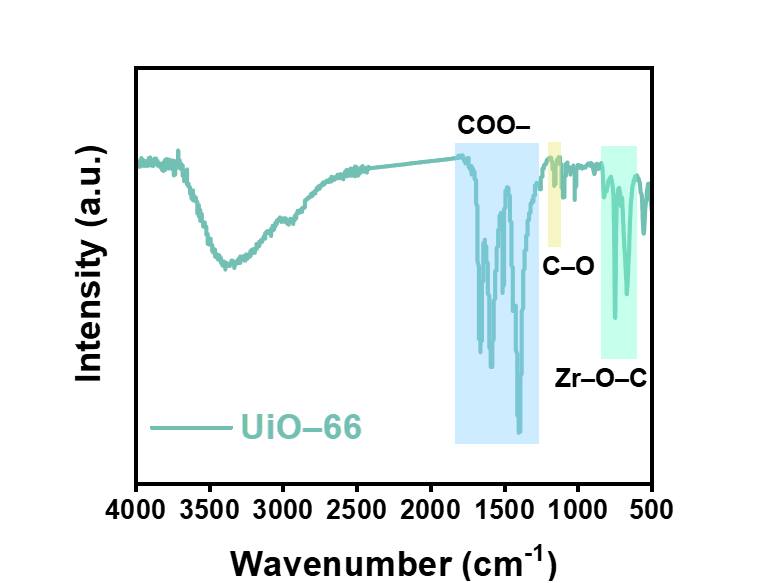


**Supplementary Figure S2.** FT–IR spectra of UiO–66.


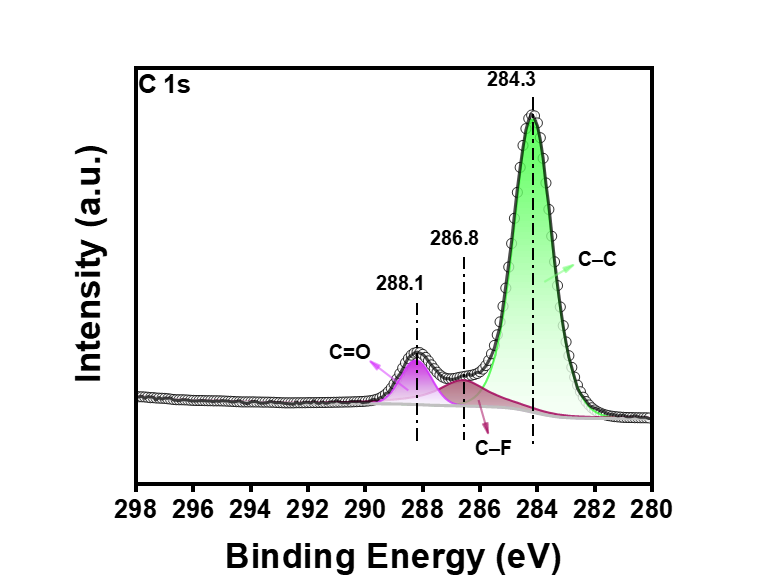


**Supplementary Figure S3.** XPS spectra corresponding to C 1s of UiO–66–F.


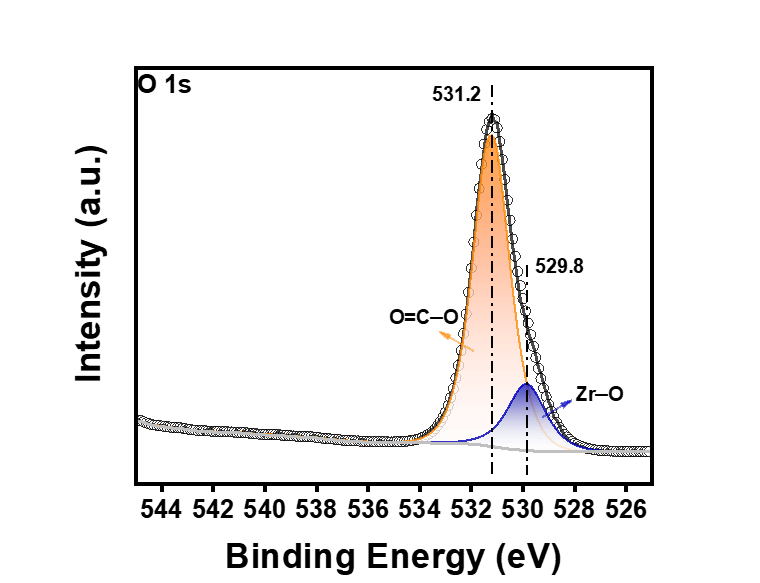


**Supplementary Figure S4.** XPS spectra corresponding to O 1s of UiO–66–F.


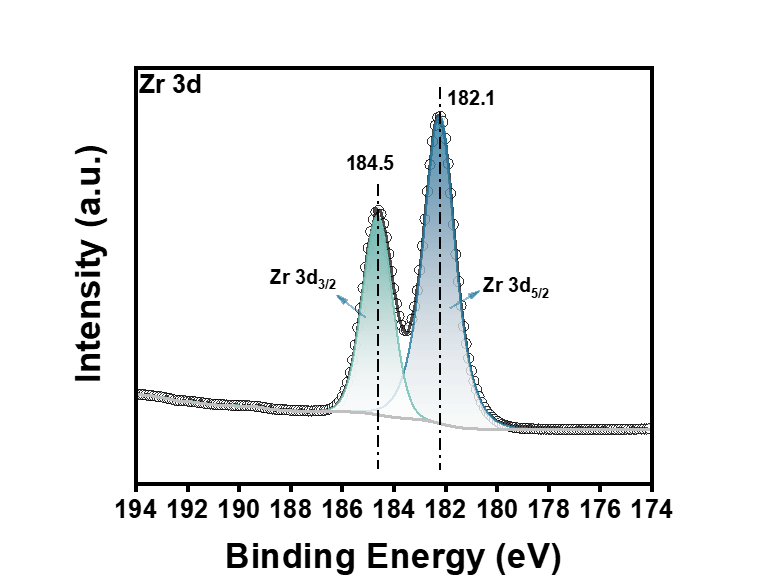


**Supplementary Figure S5.** XPS spectra corresponding to Zr 3d of UiO–66–F.


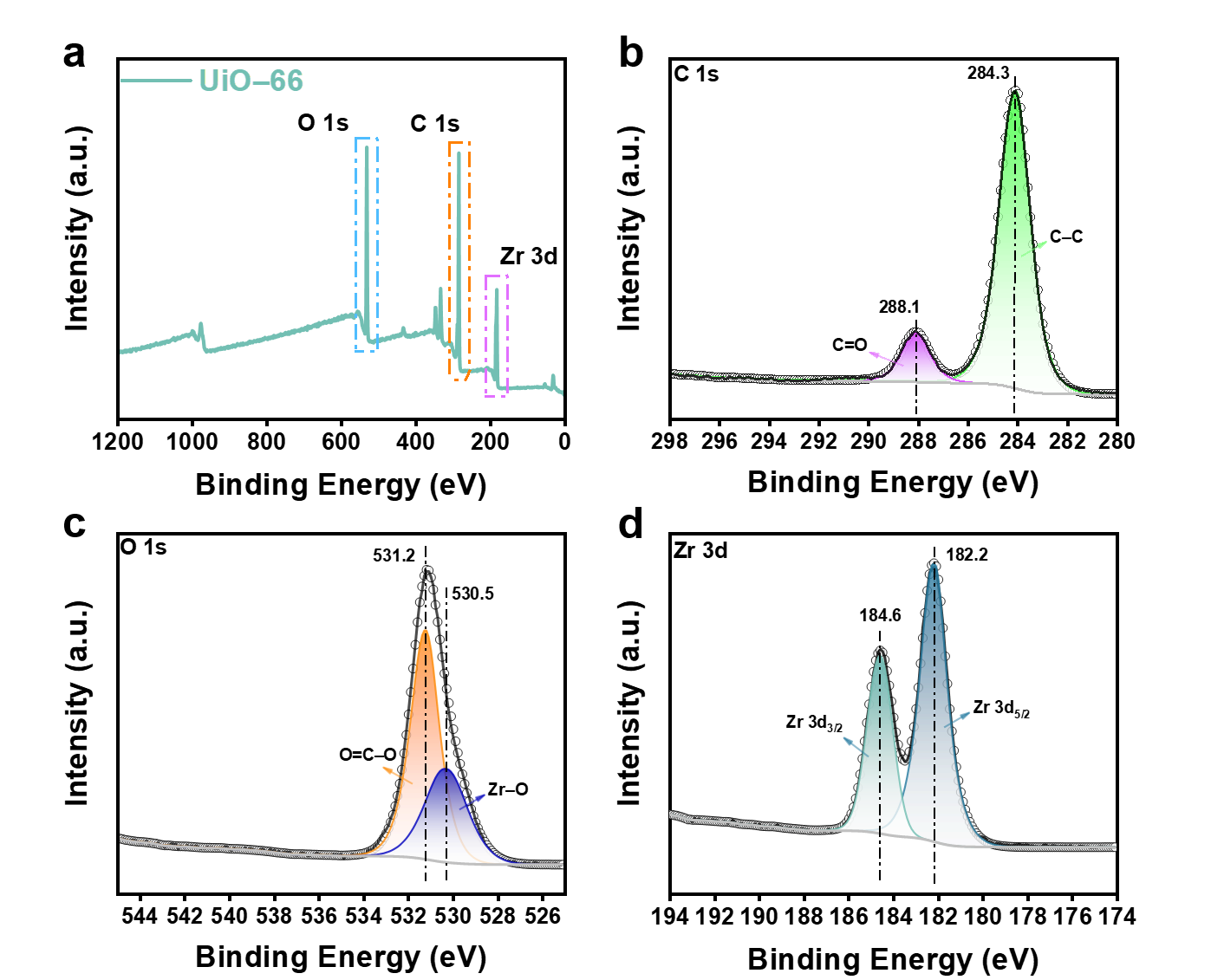


**Supplementary Figure S6.** Full XPS spectrum of UiO–66 and corresponding high-resolution C 1s, O 1s, and Zr 3d spectra.


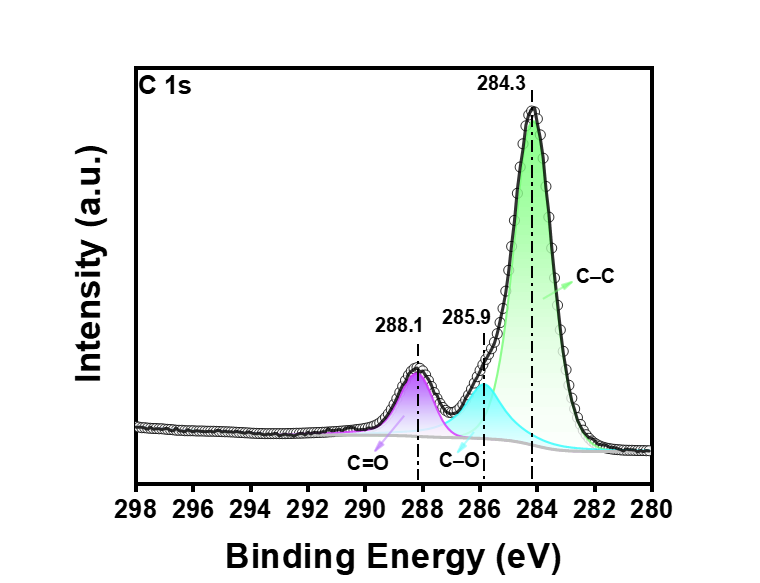


**Supplementary Figure S7.** XPS spectra corresponding to C 1s of UiO–66–OH.


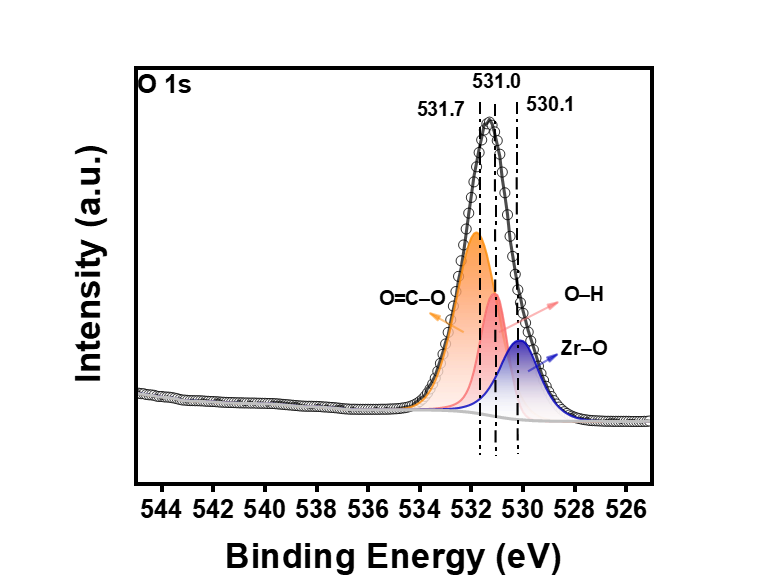


**Supplementary Figure S8.** XPS spectra corresponding to O 1s of UiO–66–OH.


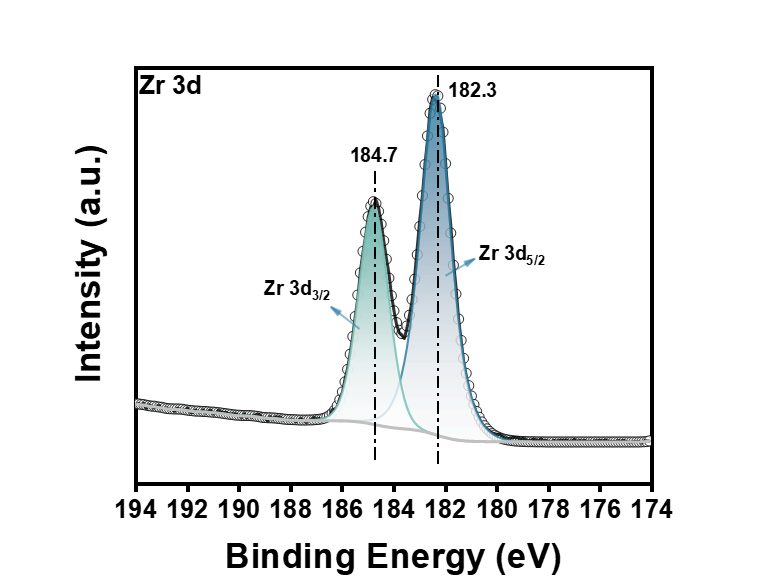


**Supplementary Figure S9.** XPS spectra corresponding to Zr 3d of UiO–66–OH.


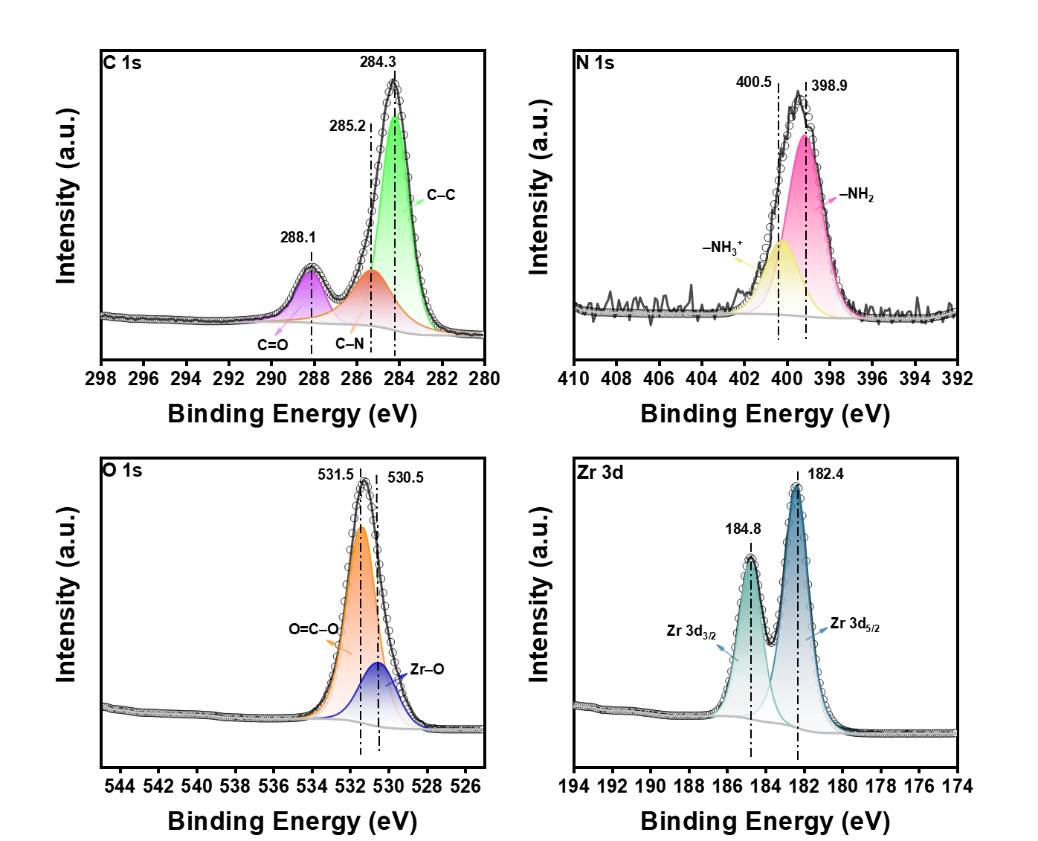


**Supplementary Figure S10.** XPS spectra corresponding to C 1s, N 1s, O 1s and Zr 3d of UiO–66–NH_2_.


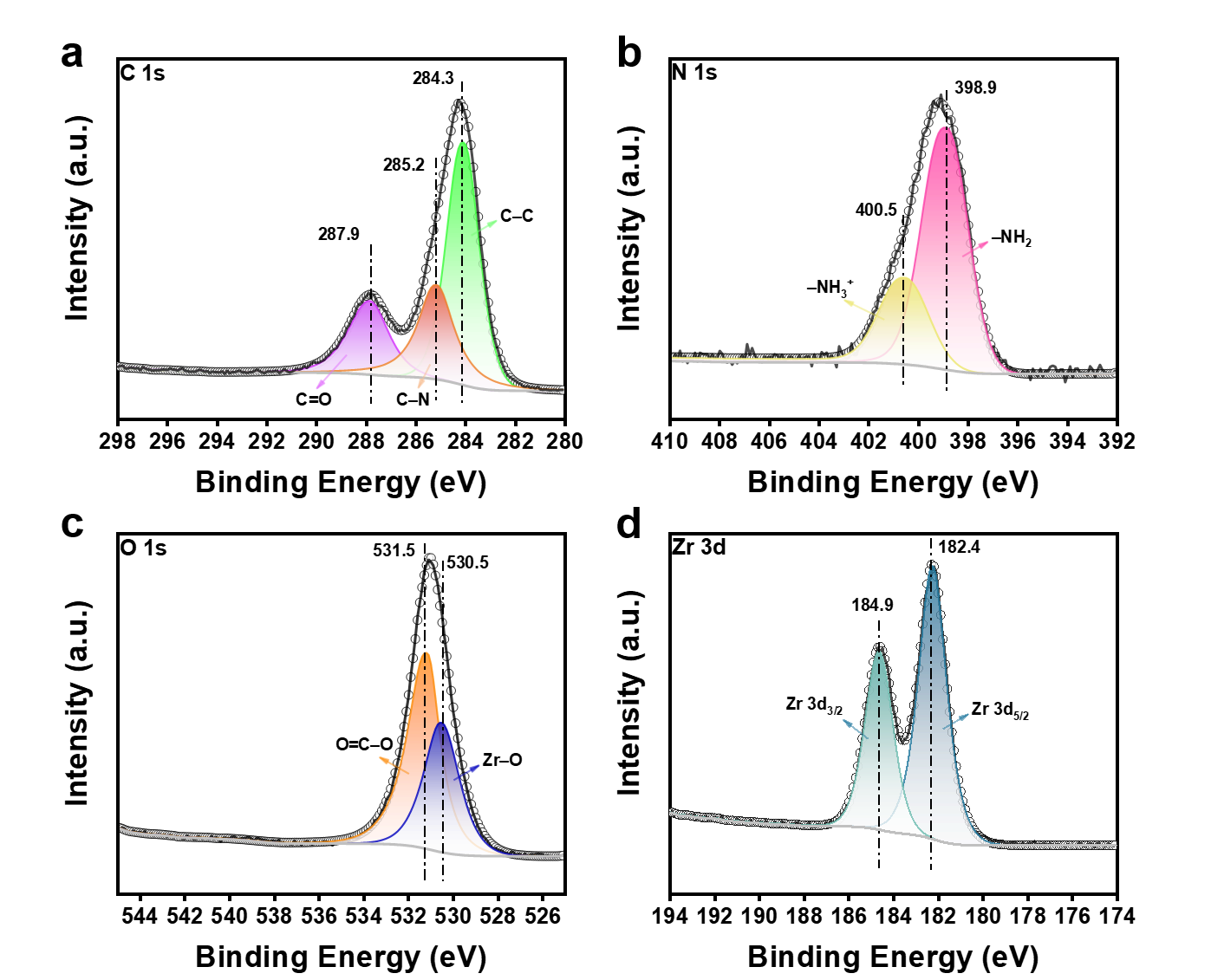


**Supplementary Figure S11.** XPS spectra corresponding to C 1s, N 1s, O 1s and Zr 3d of UiO–66–(NH_2_)_2_.


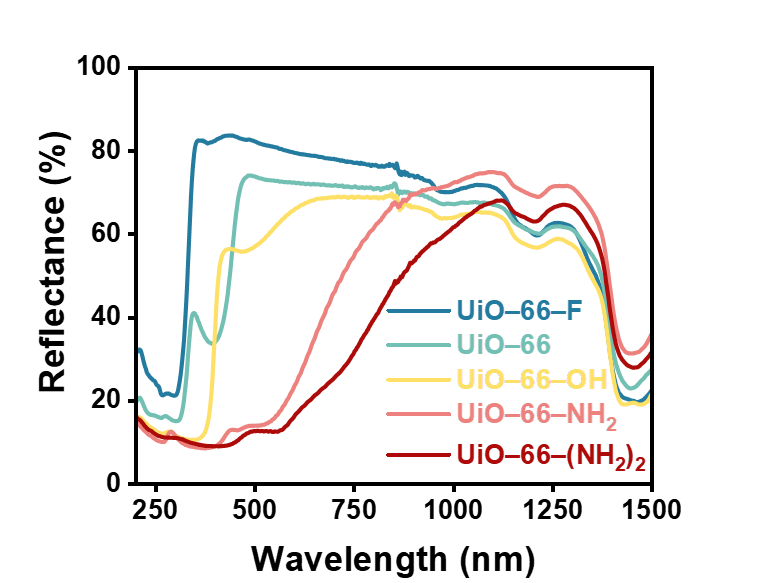


**Supplementary Figure S12.** UV–vis reflectance spectrum of UiO–66–X.


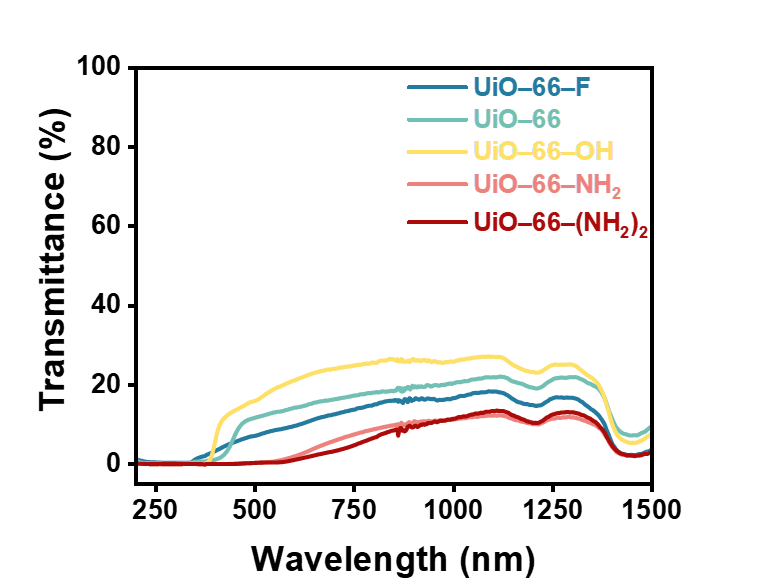


**Supplementary Figure S13.** UV–vis transmittance spectrum of UiO–66–X.


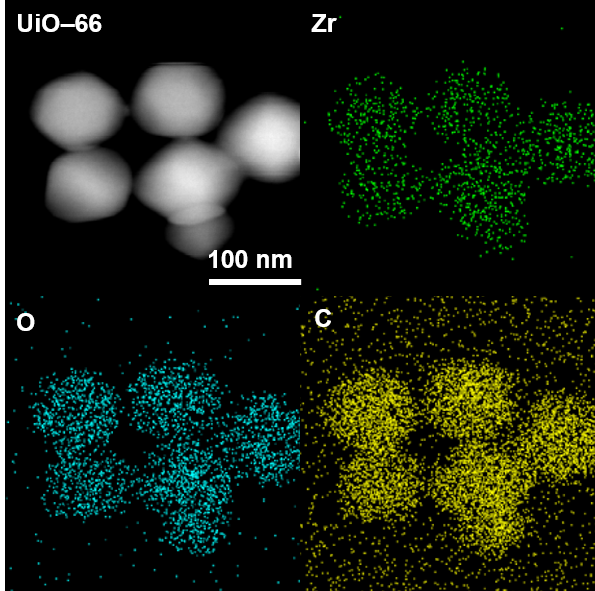


**Supplementary Figure S14.** TEM and elemental mappings of UiO–66.


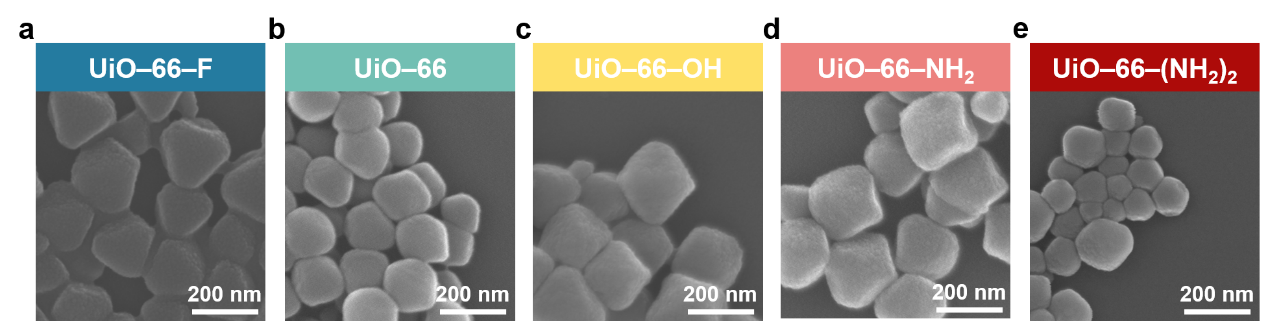


**Supplementary Figure S15.** SEM images of UiO–66–X (X=–F, –H, –OH, –NH_2_, –(NH_2_)_2_).


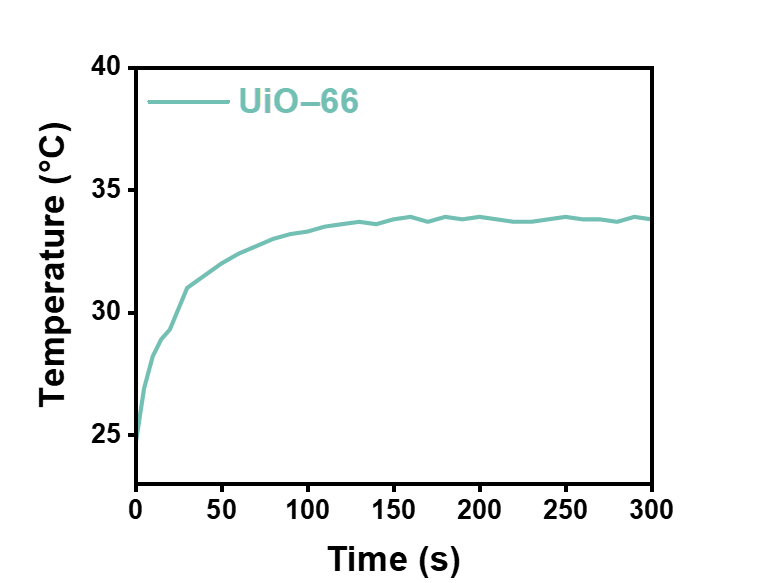


**Supplementary Figure S16.** Surface temperature variations of UiO–66 under one solar irradiation (1 kW·m^-2^) over time.


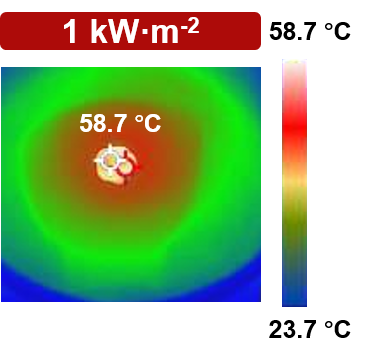


**Supplementary Figure S17.** Thermal imaging of UiO–66–(NH_2_)_2_ at 1.0 kW·m^-2^ solar intensity.


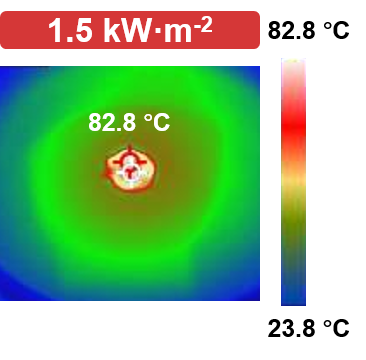


**Supplementary Figure S18.** Thermal imaging of UiO–66–(NH_2_)_2_ at 1.5 kW·m^-2^ solar intensity.


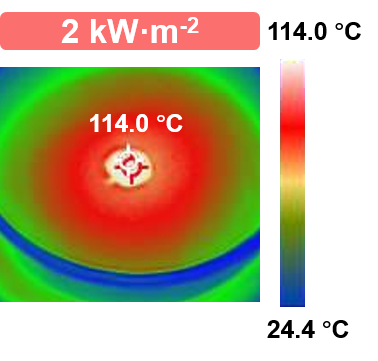


**Supplementary Figure S19.** Thermal imaging of UiO–66–(NH_2_)_2_ at 2.0 kW·m^-2^ solar intensity.


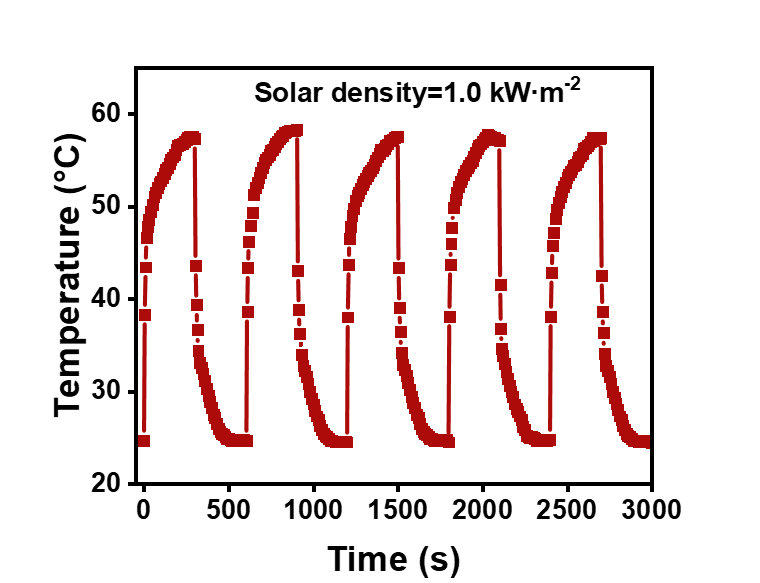


**Supplementary Figure S20.** Cyclic stability of UiO–66–(NH_2_)_2_ at one solar intensity(1.0 kW·m^-2^).


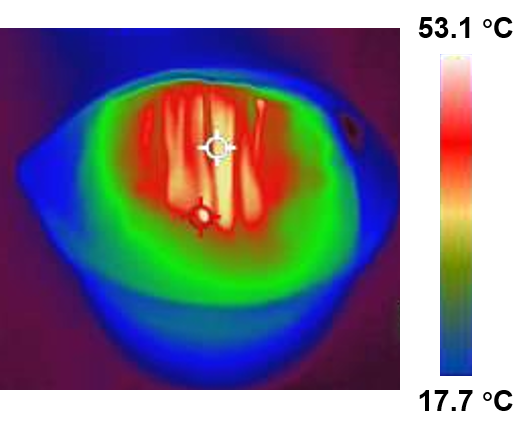


**Supplementary Figure S21.** Thermal imaging of the UiO–66–(NH_2_)_2_–paper evaporator at one solar intensity.


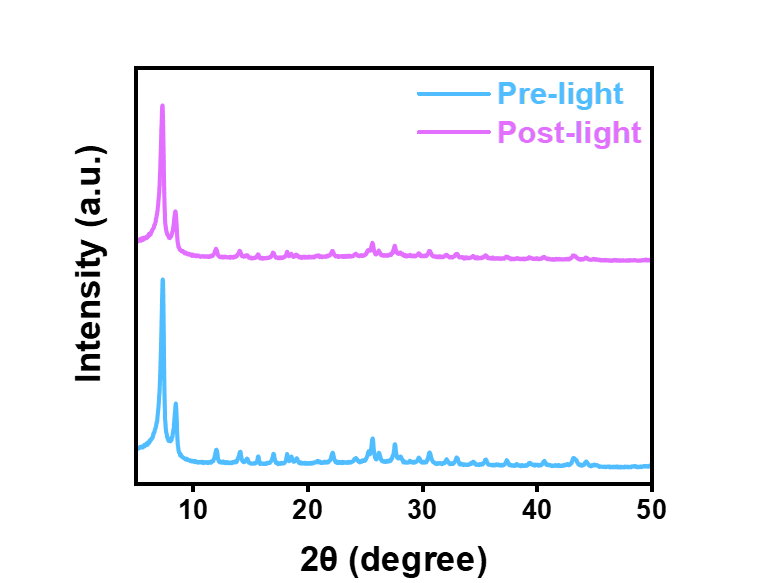


**Supplementary Figure S22.** XRD patterns of UiO–66–(NH_2_)_2_ before and after 168 h of light irradiation.


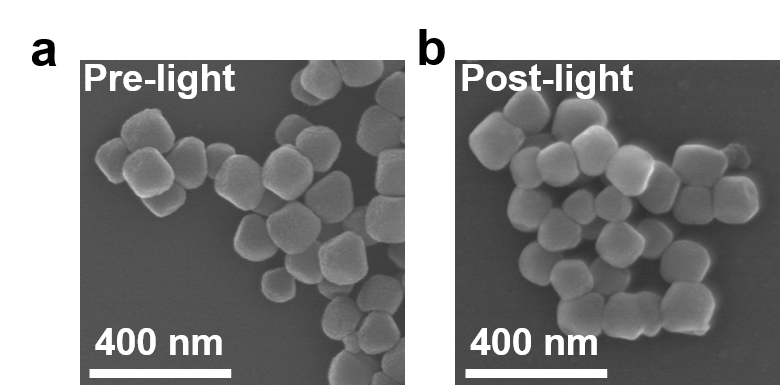


**Supplementary Figure S23.** SEM images of UiO–66–(NH_2_)_2_ before (a) and after (b) 168 h of light irradiation.

**
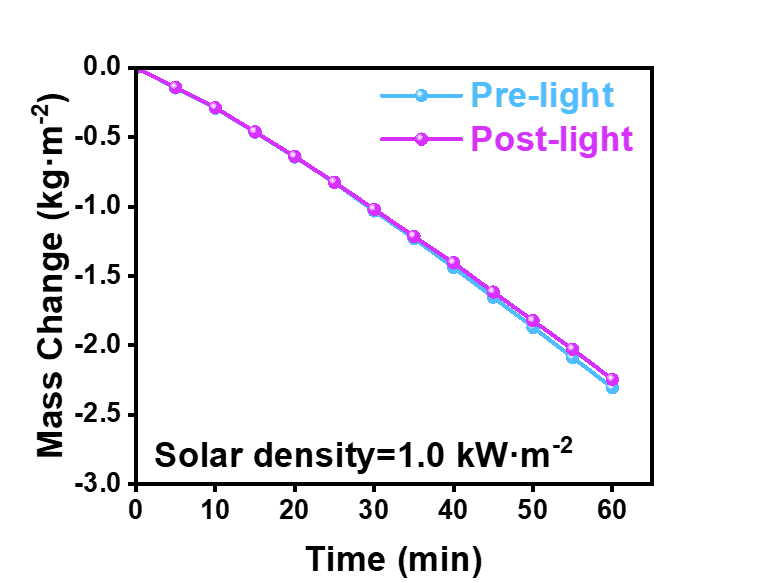
**

**Supplementary Figure S24.** Comparison of seawater evaporation performance of UiO–66–(NH_2_)_2_ before and after 168 h of light exposure under 1.0 kW·m^-2^ solar radiation.

**
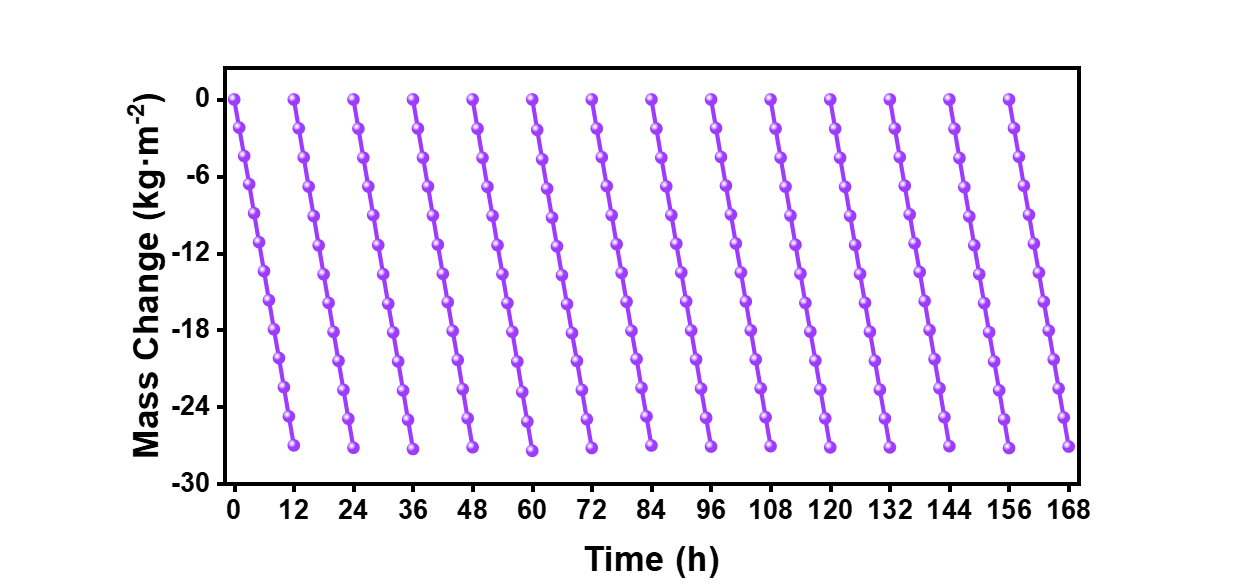
**

**Supplementary Figure S25.** Long-term seawater evaporation stability of the UiO–66–(NH_2_)_2_–paper evaporator under continuous 1.0 kW·m^-2^ solar radiation for 168 h.


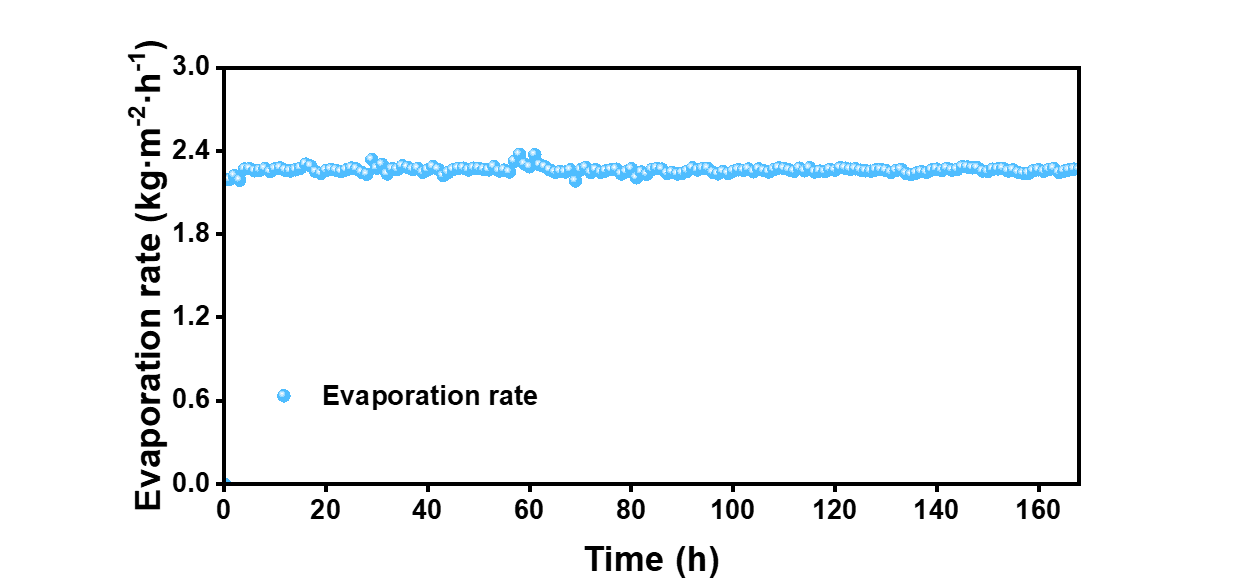


**Supplementary Figure S26.** Hourly seawater evaporation rate of the UiO–66–(NH_2_)_2_–paper evaporator under 1.0 kW·m^-2^ solar irradiation.


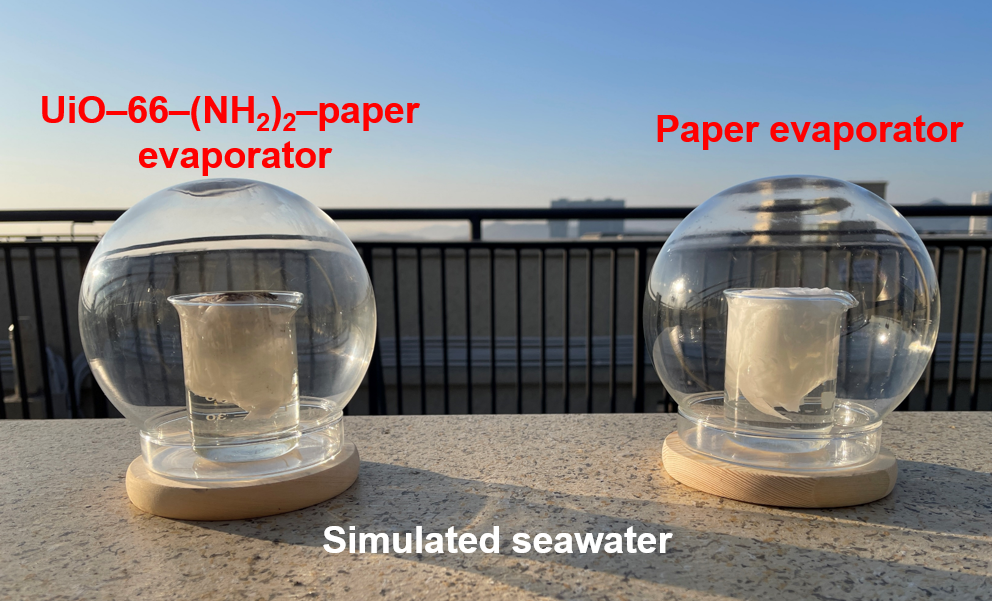


**Supplementary Figure S27.** Photographs of UiO–66–(NH_2_)_2_–paper evaporator and Paper evaporator for outdoor experiments in simulated seawater on December 17, 2024.


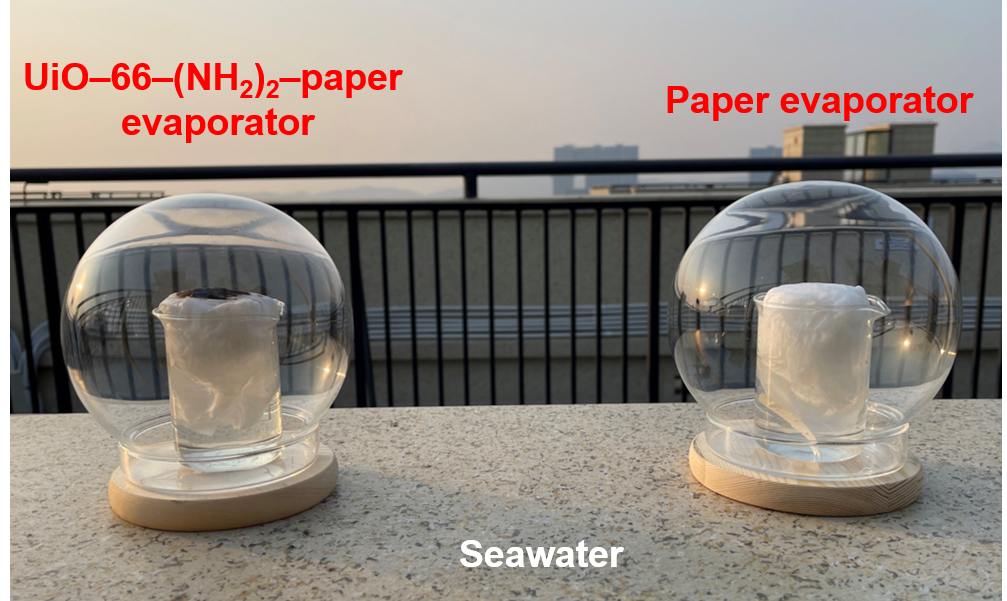


**Supplementary Figure S28.** Photographs of UiO–66–(NH_2_)_2_–paper evaporator and Paper evaporator for outdoor experiments in real seawater on December 18, 2024.


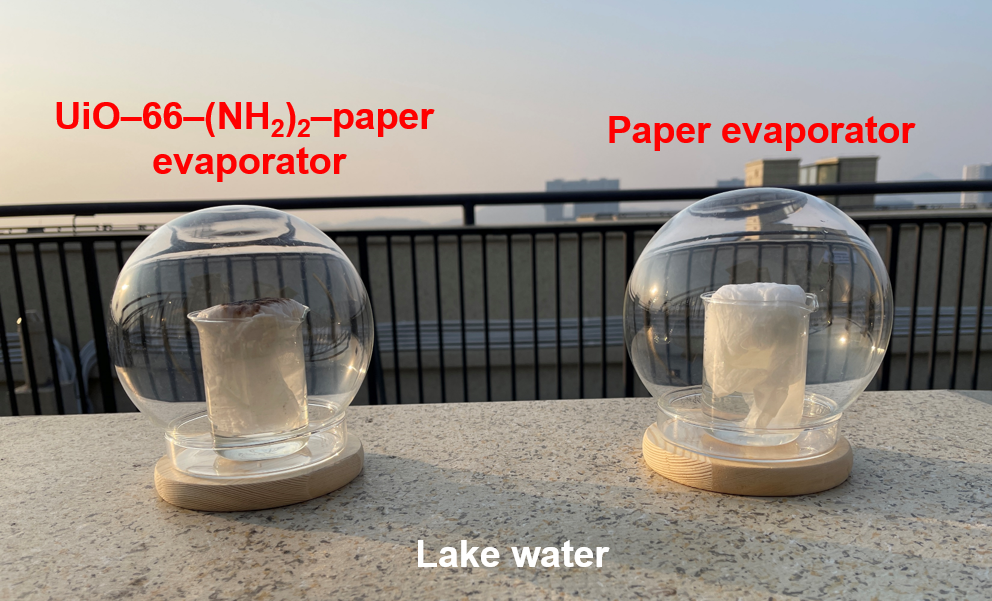


**Supplementary Figure S29.** Photographs of UiO–66–(NH_2_)_2_–paper evaporator and paper–evaporator for outdoor experiments in lake water on December 21, 2024.


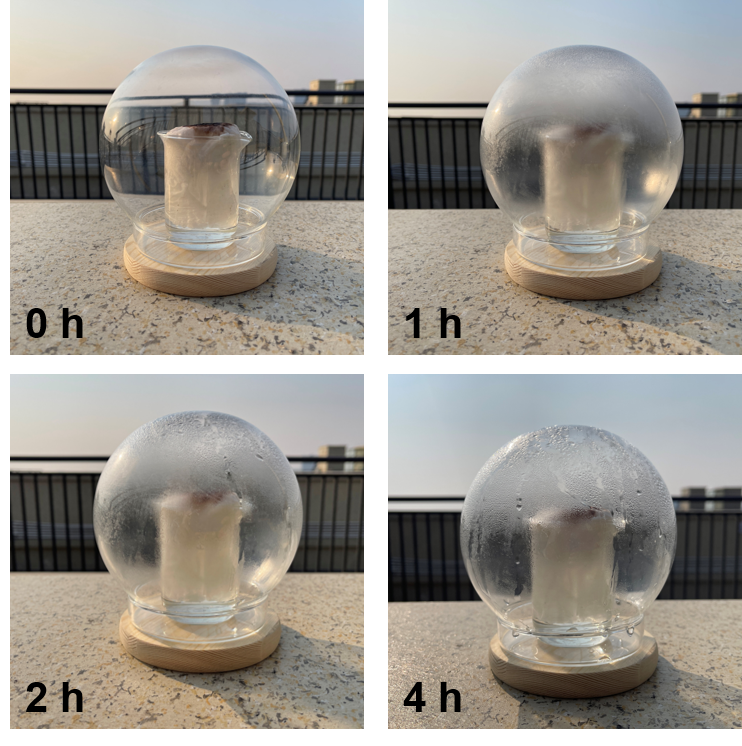


**Supplementary Figure S30.** Optical photographs of the UiO–66–(NH_2_)_2_–paper evaporator at different times of daylight.


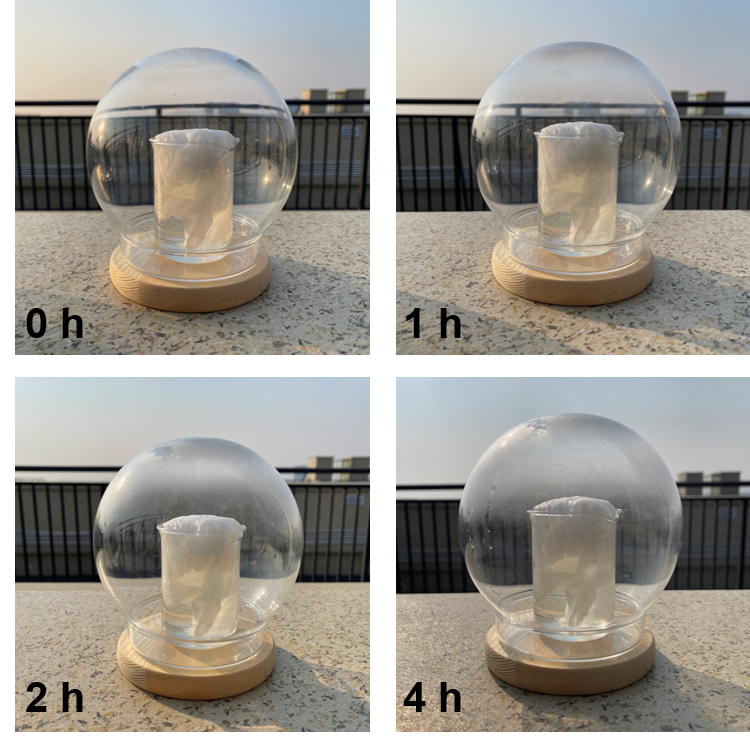


**Supplementary Figure S31.** Optical photographs of the paper–evaporator at different times of daylight.


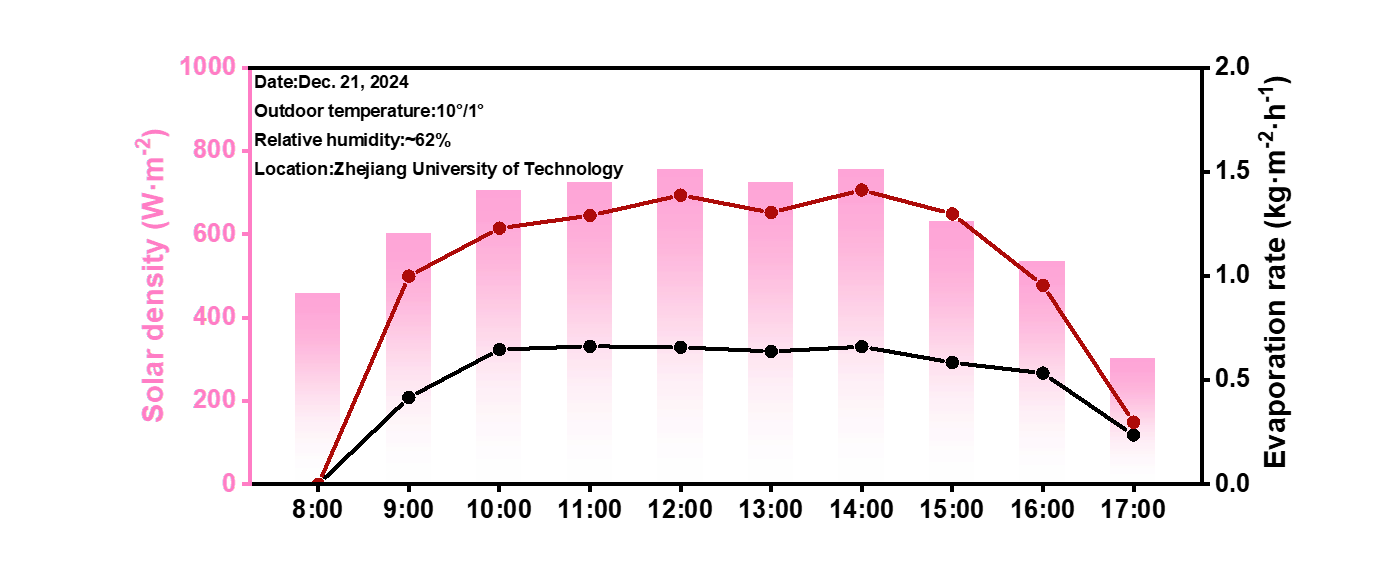


**Supplementary Figure S32.** Cyclic insolation intensity and evaporation rate of the UiO–66–(NH_2_)_2_–paper and Paper–evaporators during outdoor testing monitored by time of day on a sunny day from 8:00 to 17:00.


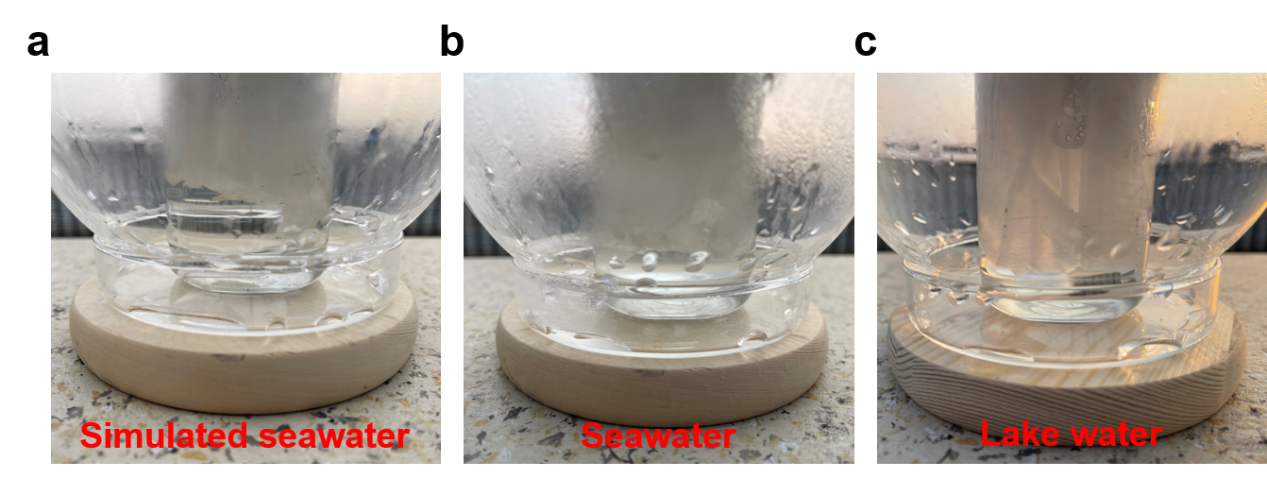


**Supplementary Figure S33.** Photograph of pure water collected after desalination of simulated seawater, real seawater and lake water by UiO–66–(NH_2_)_2_–paper evaporator.

***Supplementary Tables***

**Table S1.** Evaporation performance of reported polymer-based evaporators.

| Evaporator | Evaporation rate (kg·m^-2^·h^-1^) | Efficiency (%) | Refs. |
| --- | --- | --- | --- |
| Carbonized wood–slice | 1.45 | 91.30 | ^[4]^ |
| Aluminophosphate–treated wood | 1.42 | 90.80 | ^[5]^ |
| Carbonized longitudinal wood | 1.08 | 74.00 | ^[6]^ |
| TiN/wood-derived carbon foam | 1.47 | 92.50 | ^[7]^ |
| Carbonized moldy bread | 0.96 | 71.40 | ^[8]^ |

**Table S2.** Evaporation performance of reported carbon materials/polymer evaporators.

| Evaporator | Evaporation rate (kg·m^-2^·h^-1^) | Efficiency (%) | Refs. |
| --- | --- | --- | --- |
| NC–RD/CNF | 1.46 | 91.00 | ^[9]^ |
| Bi–C/CF | 1.50 | 91.90 | ^[10]^ |
| LC/CA/PDMS | 1.62 | 83.80 | ^[11]^ |
| PAM–CBF | 1.79 | 94.40 | ^[12]^ |
| Porous carbon /wood | 2.38 | 88.00 | ^[13]^ |
| Porous carbon/paper pulp fiber | 1.80 | 87.60 | ^[14]^ |
| 3D graphene network | 1.64 | 91.80 | ^[15]^ |
| RGO–SA–CNT aerogel | 1.62 | 83.00 | ^[16]^ |
| Snake–scale–like porous carbon | 1.33 | 91.08 | ^[17]^ |
| N–doped porous graphene | 1.50 | 80.00 | ^[18]^ |

**Table S3.** Evaporation performance of reported MOFs/polymer evaporators.

| Evaporator | Evaporation rate (kg·m^-2^·h^-1^) | Efficiency (%) | Refs. |
| --- | --- | --- | --- |
| PON@MOF | 2.14 | 98.50 | ^[19]^ |
| [Cu_2_(OH)(BTC)(H_2_O)]_n_·2H_2_O/PVDF | 1.44 | 90.80 | ^[20]^ |
| Ni_3_(HITP)_2_–paper | 2.60 | 71.10 | ^[21]^ |
| Ni–MOF/Wood | 2.07 | 91.50 | ^[22]^ |
| MIL–101/Wood | 1.81 | 89.00 | ^[23]^ |
| PANI/ZIF–8 | 1.07 | 66.80 | ^[24]^ |
| PSS@HKUST–1/SWCNT | 1.38 | 90.80 | ^[25]^ |
| PES/PDA/MOF–30 mg | 1.85 | 98.40 | ^[26]^ |
| Zr–Fc MOF/SWCNT/Gelatin | 1.53 | 95.00 | ^[27]^ |
| CFc/TiO_2_/Ti–MOF | 1.86 | 97.20 | ^[28]^ |
| PFC–771 | 1.33 | 91.08 | ^[29]^ |
| CoTCPP–Bi@PU | 1.43 | 94.00 | ^[30]^ |
| UiO–66–(NH_2_)_2_–paper | 2.34 | 97.40 | This work |

**Table S4.** Compositional information of water sources used in outdoor experiments.

| Sample | Cl^-^, mg/L | SO_4_^2-^, mg/L | Na^+^, mg/L | K^+^, mg/L | Mg^2+^, mg/L | Ca^2+^, mg/L |
| --- | --- | --- | --- | --- | --- | --- |
| simulated seawater | 24187.77 | 478.83 | 10588.51 | 399.82 | 2749.02 | 399.11 |
| real seawater | 25540.16 | 3016.66 | 10280.45 | 399.99 | 1088.75 | 814.18 |
| lake water | 52.01 | 76.01 | 31.81 | 6.92 | 7.79 | 39.85 |

***References***

[1] T. He, X. Xu, B. Ni, H. Wang, Y. Long, W. Hu, X. Wang, *Nanoscale* **2017**, *9*, 19209.

[2] G. Kresse, D. Joubert, *Phys. Rev. B* **1999**, *59*, 1758.

[3] J. P. Perdew, K. Burke, M. Ernzerhof, *Phys. Rev. Lett.* **1996**, *77*, 3865.

[4] P.–F. Liu, L. Miao, Z. Deng, J. Zhou, H. Su, L. Sun, S. Tanemura, W. Cao, F. Jiang, L.–D. Zhao, *Mater. Today Energy* **2018**, *8*, 166.

[5] T. Chen, Z. Wu, Z. Liu, J. T. Aladejana, X. (Alice) Wang, M. Niu, Q. Wei, Y. Xie, *ACS Appl. Mater. Interfaces* **2020**, *12*, 19511.

[6] H. Liu, C. Chen, G. Chen, Y. Kuang, X. Zhao, J. Song, C. Jia, X. Xu, E. Hitz, H. Xie, S. Wang, F. Jiang, T. Li, Y. Li, A. Gong, R. Yang, S. Das, L. Hu, *Adv. Energy Mater.* **2018**, *8*, 1701616.

[7] D. Guo, X. Yang, *Sci. China Mater.* **2019**, *62*, 711.

[8] Y. Zhang, S. K. Ravi, J. V. Vaghasiya, S. C. Tan, *iScience* **2018**, *3*, 31.

[9] Z. Jiang, X. He, K. Zhu, Q. An, Z. Xiao, X. Dong, L. Shao, S. Zhai, *Desalination* **2024**, *592*, 118085.

[10] J. Jiang, Y. Xu, C. Tang, X. Wang, W. Wei, L. Ai, *Desalination* **2023**, *560*, 116680.

[11] Y. Ko, S. Lee, J. Jang, G. Kwon, K. Lee, Y. Jeon, A. Lee, T. Park, J. Kim, J. You, *Adv. Funct. Mater.* **2025**, *35*, 2414576.

[12] Y. Wu, C. Ma, K. Zhu, L. Jin, L. Song, L. Li, Y. Lu, Y. Zheng, Y. Zhang, X. Zheng, S. Wu, Y. Pang, Z. Shen, S. C. Tan, H. Chen, *Energy Environ. Sci.* **2024**, *17*, 9303.

[13] N. Liu, L. Hao, B. Zhang, R. Niu, J. Gong, T. Tang, *Energy Environ. Mater.* **2022**, *5*, 617.

[14] L. Hao, N. Liu, R. Niu, J. Gong, T. Tang, *Sci. China Mater.* **2022**, *65*, 201.

[15] K. Kim, S. Yu, C. An, S.–W. Kim, J.–H. Jang, *ACS Appl. Mater. Interfaces* **2018**, *10*, 15602.

[16] X. Hu, W. Xu, L. Zhou, Y. Tan, Y. Wang, S. Zhu, J. Zhu, *Adv. Mater.* **2017**, *29*, 1604031.

[17] M. R. Singh, C. Xiang, N. S. Lewis, *Sustain. Energy Fuels* **2017**, *1*, 458.

[18] Y. Ito, Y. Tanabe, J. Han, T. Fujita, K. Tanigaki, M. Chen, *Adv. Mater.* **2015**, *27*, 4302.

[19] Y. Jiang, Q. Lu, X. Zhao, K. Zhao, A. Liu, J. Pan, *ACS Appl. Mater. Interfaces* **2024**, *16*, 59030.

[20] Z. Li, X. Ma, D. Chen, X. Wan, X. Wang, Z. Fang, X. Peng, *Adv. Sci.* **2021**, *8*, 2004552.

[21] Y. Qian, G. Xue, L. Chen, G. Xu, G. Wang, *Adv. Mater.* **2024**, *36*, 2310795.

[22] P. He, L. Hao, N. Liu, H. Bai, R. Niu, J. Gong, *Chem. Eng. J.* **2021**, *423*, 130268.

[23] S. Jing, Q. Ji, A. Wang, J. Zhao, H. Liang, F. Chen, P. Kannan, P. Tsiakaras, *Appl. Therm. Eng.* **2024**, *244*, 122629.

[24] Y. Peng, X. Wei, Y. Wang, W. Li, S. Zhang, J. Jin, *ACS Nano* **2022**, *16*, 8329.

[25] X. Ma, W. Fang, Y. Guo, Z. Li, D. Chen, W. Ying, Z. Xu, C. Gao, X. Peng, *Small* **2019**, *15*, 1900354.

[26] Y. Wang, R. Yu, *J. Environ. Chem. Eng.* **2023**, *11*, 110422.

[27] X. Ma, Z. Deng, Z. Li, D. Chen, X. Wan, X. Wang, X. Peng, *J. Mater. Chem. A* **2020**, *8*, 22728.

[28] R. Meng, Y. Lu, L. Zou, H. Du, X. Li, B. Zhu, D. K. Macharia, M. Amidpour, Z. Chen, L. Zhang, *Desalination* **2024**, *578*, 117455.

[29] Z.–Y. Wang, R. Wang, H. M. Johnson, L. Cai, A.–A. Zhang, Q. Zhang, T.–F. Liu, *J. Mater. Chem. A* **2024**, *12*, 12592.

[30] L. He, J. He, E.–X. Chen, Q. Lin, *Chem. Sci.* **2024**, *15*, 17498.
